# VCAM1: an effective diagnostic marker related to immune cell infiltration in diabetic nephropathy

**DOI:** 10.3389/fendo.2024.1426913

**Published:** 2024-09-10

**Authors:** Yuanyuan Deng, Sai Zhang, Zheng Luo, Pengfei He, Xinyu Ma, Yu Ma, Jing Wang, Liyang Zheng, Ni Tian, Shaoning Dong, Xingkun Zhang, Mianzhi Zhang

**Affiliations:** ^1^ Department of Nephrology, Tianjin Academy of Traditional Chinese Medicine, Tianjin, China; ^2^ Dongfang Hospital, Beijing University of Chinese Medicine, Beijing, China; ^3^ College of Basic Medicine, Chengdu University of Traditional Chinese Medicine, Chengdu, China; ^4^ Department of Clinical Medicine, Tianjin Medical University, Tianjin, China

**Keywords:** diabetic nephropathy, immune infiltration, bioinformatics analysis, experimental verification, biomarker

## Abstract

**Introduction:**

The role of immune cells in the pathogenesis and advancement of diabetic nephropathy (DN) is crucial. The objective of this study was to identify immune-cell-related biomarkers that could potentially aid in the diagnosis and management of DN.

**Methods:**

The GSE96804 dataset was obtained from the Gene Expression Omnibus (GEO) database. Then, screen for intersections between differentially expressed genes (DEGs) and immune-related genes (IRGs). Identify core genes through protein-protein interaction (PPI) networks and the Cytoscape plugin. Subsequently, functional enrichment analysis was conducted. In addition, ROC analysis is performed to accurately identify diagnostic biomarkers. Apply the CIBERSORT algorithm to evaluate the proportion of immune cell infiltration. Finally, the mRNA, protein, and immunofluorescence expression of the biomarker was validated in the DN rat model.

**Results:**

The study yielded 74 shared genes associated with DN. Enrichment analysis indicated significant enrichment of these genes in focal adhesion, the humoral immune response, activation of the immune response, Cytokine-cytokine receptor interaction, and IL-17 signaling pathway. The optimal candidate gene VCAM1 was identified. The presence of VCAM1 in DN was further validated using the ROC curve. Analysis of immune cell infiltration matrices revealed a high abundance of monocytes, naïve B cells, memory B cells, and Macrophages M1/M2 in DN tissues. Correlation analysis identified one hub biomarker associated with immune-infiltrated cells in DN. Furthermore, our findings were validated through *in vivo* RT qPCR, WB, and IF techniques.

**Conclusions:**

Our research indicates that VCAM1 is a signature gene associated with DN and is linked to the progression, treatment, and prognosis of DN. A comprehensive examination of immune infiltration signature genes may offer new perspectives on the clinical diagnosis and management of DN.

## Graphical Abstract

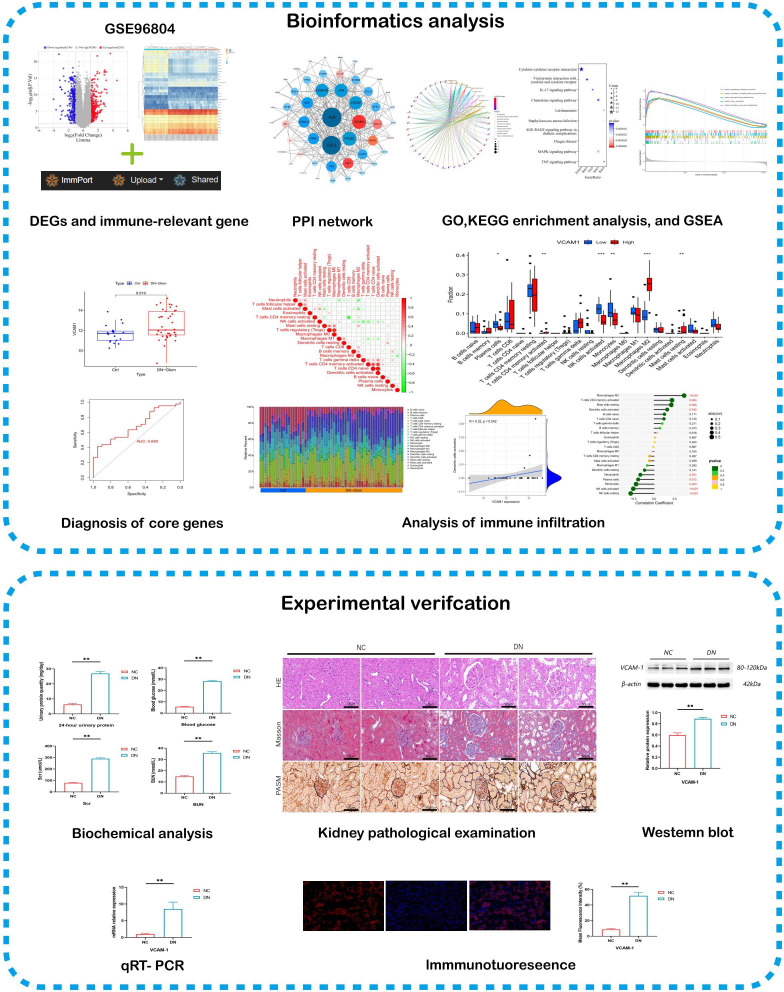

## 1 Introduction

Diabetic nephropathy (DN), a prevalent and severe microvascular complication of DM, is recognized as a leading factor in end-stage renal disease (1, 2). The principal clinical indicators of DN include sustained albuminuria and progressive decline in renal function (3), while characteristic pathological features encompass thickening of the glomerular basement membrane, mesangial expansion, nodular sclerosis, and marked glomerulosclerosis (4). The aetiology of microvascular complications in diabetes is intricate and remains incompletely elucidated. Moreover, a substantial body of research indicates that chronic inflammation and infiltrating immune cells are significant factors in the development and advancement of DN (5).

Currently, the diagnosis of DN is primarily based on the presence of microalbuminuria and a gradual decrease in renal function. However, it has been observed that some patients with microalbuminuria/proteinuria do not exhibit a decline in renal function, specifically in terms of glomerular filtration rate (6). This suggests that relying solely on detecting proteinuria and glomerular filtration rate is inadequate for accurately diagnosing and predicting the progression of early DN (7). Research indicates that oxidative stress, inflammatory responses, and activation of the renin-angiotensin-aldosterone system constitute major pathogenetic features of DN (8). Furthermore, contemporary studies have demonstrated a strong association between chronic inflammatory and immune disorders and DN (9). The immunopathogenesis of DN involves multiple cytokines and signal transduction pathways (10). A critical factor in the progression of inflammation, apoptosis, and fibrosis in DN is the imbalanced phenotypic activation of macrophages (5, 11). In patients with diabetes mellitus (DM), wound healing is characterized by dysregulated and persistent M1 (pro-inflammatory) macrophage polarization. In contrast, normal wound healing typically involves a transition to M2 (pro-healing) macrophages around the third day post-injury (12). DN is primarily modulated by T cells, with an imbalance between helper T cell 17 (Th17) and regulatory T cell (Treg) subsets playing a crucial role in the pathogenesis of DN (13, 14). There is substantial evidence indicating significant alterations in the pattern of immune infiltration within the glomeruli of DN patients (15). Podocyte damage is identified as the primary factor contributing to the development of proteinuria in this condition. Additionally, indoleamine 2,3-dioxygenase 1 (IDO1), lymphocyte-specific protein tyrosine kinase (LCK), and hematopoietic cell kinase (HCK) have been identified as novel immune-related markers in DN patients (16, 17). Moreover, extensive research has established the presence of foot cell damage in the initial phases of DN and its significant contribution to disease advancement (18). Consequently, investigating biomarkers that target glomerular injury in DN can provide timely insights into renal structure and dysfunction in individuals with DM, thereby facilitating early detection, prognosis, and prevention of DN.

Hence, it is imperative to investigate the mechanism by which podocyte injury leads to immune infiltration, as this aspect has not been thoroughly explored in relation to the pathogenesis of DN. This study represents the pioneering effort to elucidate the specific role of immune infiltration in DN by integrating DN microarray data with immune infiltration genes. The flowchart of this study is shown in the Graphical Abstract.

## 2 Materials and methods

### 2.1 GEO datasets acquisition and preprocessing

The GSE96804 dataset of DN was retrieved from the Gene Expression Synthesis (GEO) database (http://www.ncbi.nlm.nih.gov/geo/) (19), comprising 19 normal kidney glomeruli samples and 41 diabetes kidney glomeruli samples (**Supplementary Table S1**). Utilizing the Bioconductor package in R software (4.2.2, https://www.r-project.org/) (20), gene expression matrices were generated by utilizing probe ID and Affy packages to standardize the data. The R software package was then utilized to identify Differentially Expressed Genes (DEGs) (21). The “limma” R package was utilized with thresholds of p < 0.05 and | log2 FC | > 1 to identify DEGs. Visualization of the DEGs was performed using the “ggplot2” and “pheatmap” R packages.

### 2.2 Protein-protein interaction establishment and identification of hub genes

A total of 1793 genes associated with immunity were acquired from the Imm-Port Shared Data website (https://www.immport.org/shared/genelists) (22). The identification of overlapping genes between the DEGs and the immune-relevant gene list was achieved through the use of Venn diagrams generated with R software. The shared objectives were then added to the STRING database (https://string-db.org/) (23) to generate a Protein-protein interaction (PPI) Network for the ‘Homo sapiens’ species, with confidence scores of 0.400 or above. Afterwards, the findings were retrieved from the STRING web database and then transferred to Cytoscape 3.8.2 (24) for the creation of a visual representation of a molecular interaction network. The hub genes in our constructed PPI network were identified using the Maximal Clique Centrality (MCC) and Degree algorithms within the CytoHubba plug-in of Cytoscape software.

### 2.3 Biological function and pathway analysis

Gene Ontology (GO) and Kyoto Encyclopedia of Genes and Genomes (KEGG) pathway enrichment analyses were conducted using R software. The “ggplot,” “enrichplo,” and “clusterProfiler” packages facilitated these evaluations, with a significance threshold established at *p* < 0.05. Results were subsequently visualized for interpretation.

### 2.4 Single gene GSEA

Gene Set Enrichment Analysis (GSEA) (25, 26) was employed to examine the signaling pathways linked to the central hub in DN, with phenotypes determined by the expression levels of target genes as indicated by the GSE96804 dataset. The GSEA enrichment analyses were visualized using the “limma,” “enrich plot,” and “clusterProfiler” packages in the R software. Significant enrichment was considered when the analysis results met the conditions of |NES| > 1, False discovery rate (FDR) < 0.25, and p. adjust < 0.05.

### 2.5 Diagnostic value of biomarkers in DN

In order to evaluate the predictive efficacy of the identified biomarkers, we analyzed the mRNA expression data from the GSE96804 dataset. A ROC curve was created to evaluate how well the biomarkers could differentiate between the NC and DN samples using the “pROC” package in the R software. Subsequently, to determine the trustworthiness of the identified biomarkers, the AUC values were calculated for the ROC curve area. Higher AUC values are indicative of superior predictive performance, with values approaching 1 suggesting heightened discriminatory ability between control and DN samples. The validation cohorts GSE30528 and GSE30529 were similarly assessed, utilizing mRNA expression data from these datasets to construct ROC curves and compute AUC values in order to confirm the prognostic utility of the identified biomarkers across these independent datasets.

### 2.6 Immune cell infiltration studies by subtype

The CIBERSORT algorithm (https://cibersortx.stanford.edu/) (27) was used to evaluate the frequency of invading immune cells in gene expression profiles of DN. This computational tool facilitated the estimation of infiltration levels of 22 immune cell subtypes utilizing a reference set known as LM22 in conjunction with 1000 alignments. Subsequent correlation analysis and visualization of the 22 infiltrating immune cells were conducted using the R package “complot.” Next, In R, we performed a principal component analysis (PCA) to examine the distribution of immune cell infiltrates in the DN and control groups, with the “ggplot2” package used to visualize the results. Additionally, correlations among the 22 infiltrating immune cells were depicted in a heat map.

### 2.7 Correlation analysis of identified genes and immune cells

The correlation between the specific genetic markers and the level of invading immune cells was examined using Spearman’s rank correlation analysis in the “ggpubr” package of R software. The correlations that emerged were illustrated using the visual aids offered by the “ggplot2” package.

### 2.8 Animal experimentation protocols

Twenty male Sprague Dawley (SD) rats, aged six weeks and weighing 200 ± 20g, were obtained from Beijing Vital River Laboratory Animal Technology Co., Ltd. under license SYXK (Beijing) 2019-0013. The rats were specific pathogen-free (SPF). The animals were maintained in Dongfang Hospital’s specialized pathogen-free facility at Beijing Medical University, China. The environment was regulated at 22°C with 50% relative humidity and a consistent 12-hour light-dark cycle; the rats had ad libitum access to food and water. After one week of acclimatization, the rats were randomly assigned to two groups: one group of 10 rats received a standard diet, while the other group of 10 rats received a high-sugar, high-fat diet (HSHFD, 67% maintenance feed + 10% lard + 20% sucrose + 2.5% cholesterol + 0.5% sodium cholate). Following a period of 6 weeks, rats in the high-sugar, high-fat diet group were intraperitoneally injected with Streptozocin (STZ, 35 mg/kg) dissolved in a sodium citrate buffer (0.1mol/L, pH 4.5) obtained from Solarbio. The control group of rats received only the buffer solution. Seventy-two hours following intraperitoneal injection, all rats underwent random blood glucose testing via tail-tip vein for three consecutive days. One week later, 24-hour urine samples were collected for protein quantification, with DN modeling criteria defined as a random blood glucose concentration ≥ 16.7 mmol/L (300 mg/dL) and 24-hour urine protein quantification ≥ 30 mg/24 hours for three consecutive days (28). All experimental protocols adhered to international standards for laboratory animal care as approved by the Animal Protection and Ethics Committee of Dongfang Hospital, affiliated with Beijing University of Chinese Medicine (Ethical Review No. DFYY202102R).

The rats were regularly checked for health and weight, had blood glucose tests every four weeks, and urine samples were collected at the end of the 20-week experiment. Rats were then anaesthetized with 1% sodium pentobarbital (40 mg/kg), and blood was drawn from the abdominal aorta. Subsequently, the plasma was separated, and the animals were euthanized. The blood was allowed to stand for three hours before being centrifuged again at 3500 rpm for 10 minutes at four °. The supernatant was stored at -80 °, and the kidneys were removed after blood sampling. The right kidney was weighed, and a portion of the upper section of both kidneys was preserved in 4% paraformaldehyde for 48 hours, followed by dehydration, paraffin embedding, and sectioning. Additionally, a segment from the middle of both kidneys was fixed in 2.5% glutaraldehyde and maintained at four °. The remaining kidney tissues were stored in a freezer at -80 ° for future use.

### 2.9 Chemical and reagents

A urea nitrogen (BUN) test kit (Nanjing Jiancheng, China, C013-2-1), a blood creatinine (Cr) determination kit (Nanjing Jiancheng, China, C011-2-1), a urine protein kit (Nanjing Jiancheng, China, C035-2-1), an hematoxylin-eosin (HE) staining kit (Solebao, China, G1120), a Masson’s trichrome (MASSON) staining kit (Solebao, China, G1006), a silver-silver-methylamine-periodate (PASM) Staining Kit (Solebao, China, G1790), Real-time PCR Kit (TransGen Biotech, China, AQ131-01), Streptozocin (Sigma-Aldrich, German, S0130-1G), a sodium citrate buffer (Solarbio, China, C1013), polyvinylidene fluoride (PVDF) membrane (Millipore, USA, IPVH00010), a VCAM1 antibody (Affinity, USA, DF6082), a sodium pentobarbital (Sigma, German), β-actin (Proteintech, China, 66009-1-1g), -80°C ultra-low temperature refrigerator (THermoFisher, USA), Centrifuge (Eppendorf, centrifuge 5415R).

### 2.10 Biochemical evaluation

The supernatant from the plasma was gathered for subsequent analysis. The BUN levels were determined via the Urea Assay Kit (urease method), while serum creatinine (Scr) levels were assessed using the Cr Assay kit (sarcosine oxidase method). In the 18th week of the study, SD rats were placed in metabolic cages for 24 hours to collect urine samples. The collected urine samples were subsequently mixed thoroughly and centrifuged at a speed of 3500 revolutions per minute for 10 minutes at a temperature of 4°. The resulting supernatant was meticulously aspirated, and the quantitative analysis of the 24-h-urine protein levels was conducted using a urinary protein detection kit employing the Urine protein test kit (CBB method).

### 2.11 Histological analysis

Kidney tissue samples were acquired and preserved in a 4% paraformaldehyde solution. Subsequently, the preserved tissue samples were embedded in paraffin to enable subsequent processing. Kidney sections, measuring 3 µm in thickness, were then prepared for staining utilizing a variety of techniques, including HE, PASM, and MASSON.

### 2.12 Real-time PCR

To isolate RNA from kidney tissue, the protocol outlined in the reagent manual should be followed. Subsequently, the extracted RNA should be reverse transcribed into cDNA using the steps provided in the reverse transcription kit. A reaction mixture with a total volume of 10 μL (as specified in **Supplementary Table S** **2**) should be prepared. The reaction steps can be found in **Supplementary Table S** **3**. The internal reference, GAPDH, can be utilized to determine the target mRNA expression level through the 2-ΔΔCT method. The primer sequence can be obtained from **Supplementary Table S** **4**.

### 2.13 Western blot analysis

The histone of the kidney tissue was extracted utilizing RIPA lysate, and the protein sample was quantified employing the BCA method. Subsequently, the protein was denatured following the instructions provided for the 5x protein loading buffer. Following the addition of the protein to the Sodium dodecyl sulfate-polyacrylamide gel electrophoresis (SDS-PAGE) well, the electrophoresis process was set to 60V for 30 minutes during the initial step and 100V for 60 minutes during the subsequent step. The protein was then transferred to a PVDF membrane. Upon completion of the transfer, the membrane was immersed in a prepared solution containing 5% skimmed milk powder and sealed with an additional layer of 5% skimmed milk powder for 2 hours. The samples were incubated overnight at 4°C with primary antibodies VACM1 (1:500, Abcam, DF6082) and β-actin (1:10000, Proteintech, 6009-1-1). Subsequently, they were incubated with the corresponding rabbit secondary antibody at room temperature for one hour. The chemiluminescence reaction was then performed using a hypersensitive ECL reagent kit, and the resulting images were captured using a Gel imaging analysis system.

### 2.14 Immunofluorescence (IF)

Tissue sections were fixed in 4% paraformaldehyde and then incubated overnight in a humidified chamber at 4°C with primary antibodies targeting VCAM1 (1:500). Following this, the sections underwent incubation with secondary antibodies for immunofluorescence at room temperature, shielded from light. To facilitate visualization of nuclei within kidney tissues, the sections were briefly fixed for 5 seconds in a solution of DAPI at a concentration of 2.5 µg/mL. A Nikon fluorescence microscope was used to examine the staining using a secondary antibody labeled with horseradish peroxidase.

### 2.15 Statistical analyses

Each experiment was iterated independently at least three times before an average was determined using SPSS 20.0 software (SPSS, USA) and R software (V. 4.2.2). The results were expressed as mean + standard deviation (SD). The student’s t-test was used to compare two groups, and the one-way ANOVA was utilized to compare multiple groups. A significance level of *P* < 0.05 was selected.

## 3 Results

### 3.1 Identification of DEGs between sepsis and control

**Figure 1:**
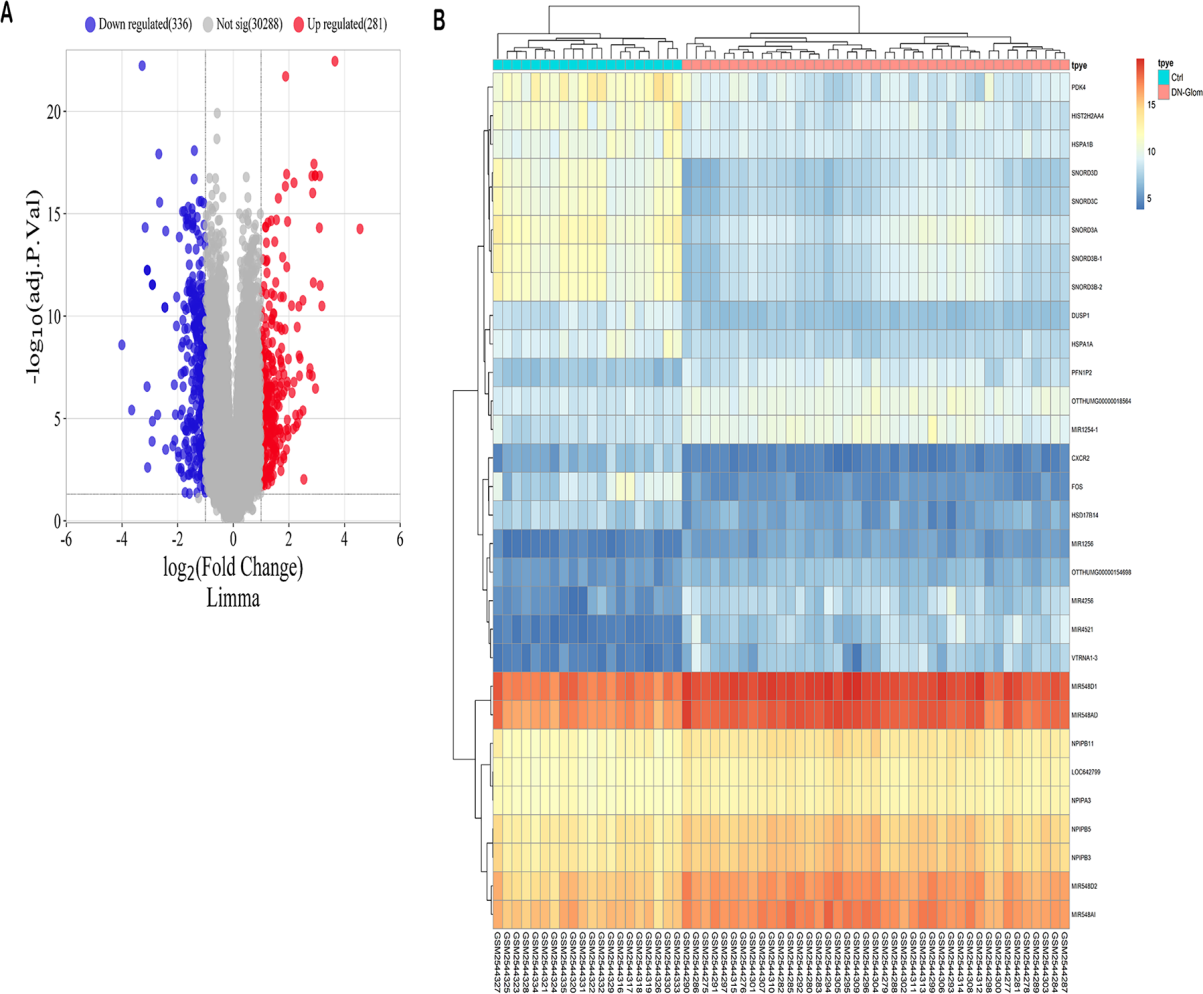
Forecasted outcomes for target genes of DN. **(A)** The volcano plot displays DEGs with consistency from GSE96804 (red dots indicate genes that are up-regulated, blue dots indicate genes that are down-regulated, and grey dots indicate genes that are not differentially expressed). **(B)** The heatmap displays the clustering analysis of DEGs with consistency in GSE96804.

The study identified a total of 610 DEGs through analysis of the GSE96804 microarray dataset using the R package “limma.” Among these DEGs, 280 genes were up-regulated in 41 cases of DN compared to 20 normal controls, while 330 genes were down-regulated (**Supplementary Table S** **5**). A volcano plot was generated to visually represent the distribution of DEGs, with genes exhibiting a log fold change greater than 0 considered upregulated and those with a log FC < 0 considered downregulated in the study group (**Figure 1A**). Furthermore, the expression patterns of these DEGs were displayed in a heat map (**Figure 1B**), providing a clear visual representation of their relative expression levels across the DN cases and normal controls.

### 3.2 PPI network analysis and hub gene screening

**Figure 2:**
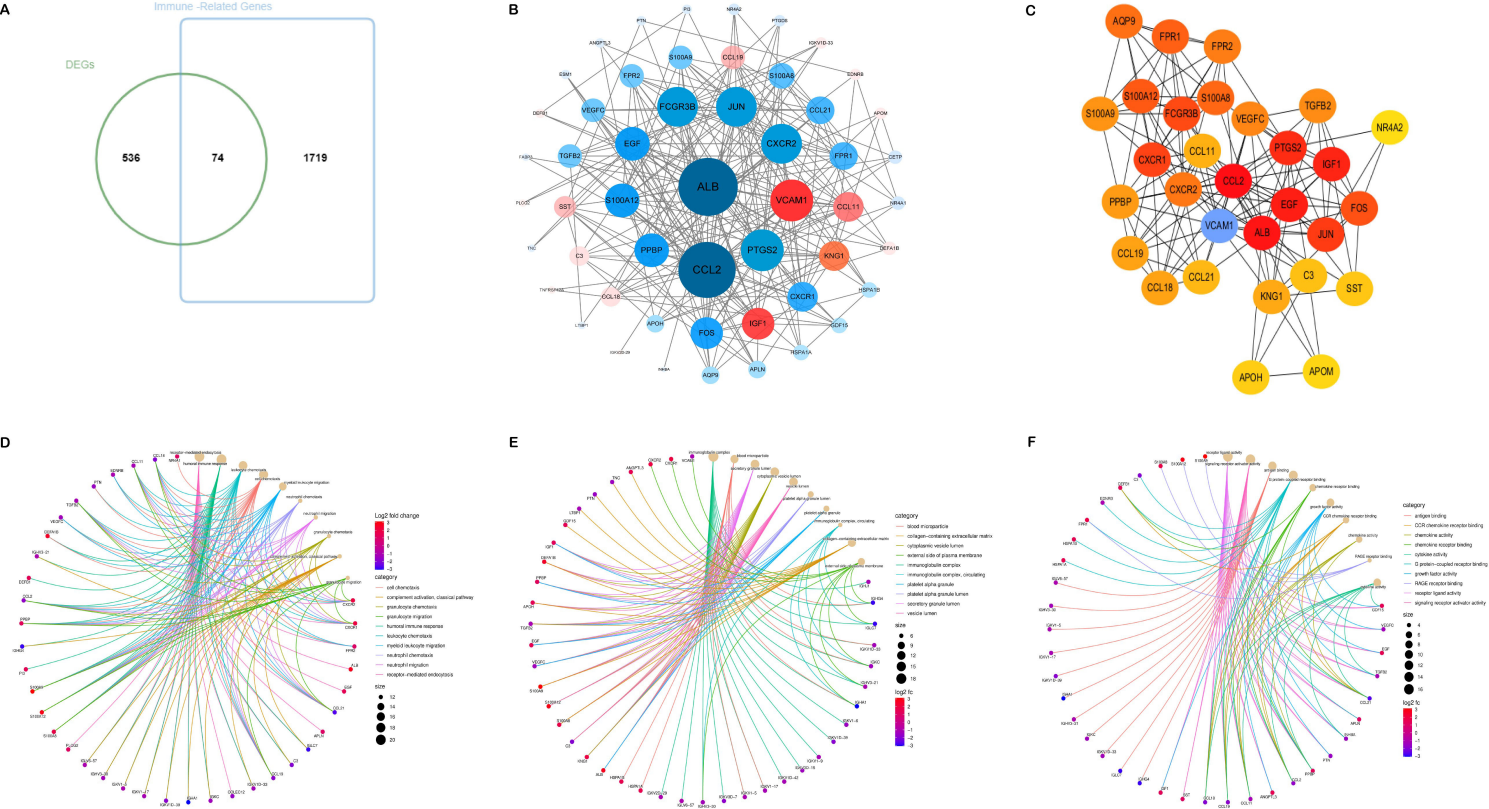
Acquisition and screening of hub immunity genes. **(A)** Venn diagram of DEGs and immune-related gene list. **(B)** PPI networks for immune-related DEGs. (Red means up-regulation, and blue means down-regulation; the darker the color and the more lines indicate a higher degree value and a more significant role in the network). **(C)** The top 30 hub immune genes were identified using the MCC algorithm to get the PPI networks. **(D–F)** represent the chord diagrams of BP, CC, and MF, respectively.

The Venn diagram presented in **Figure 2A** illustrated the discovery of 74 shared target genes linked to immune-related genes and DN. In order to enhance understanding of the diverse biological functions and mechanisms of immune infiltration and DN, proteins lacking any interaction relationships were removed from the STRING database, leading to the formation of a PPI network comprising 52 nodes and 2,299 edges (**Figure 2B**). To pinpoint the most crucial nodes in the PPI network, MCC offered by the Cytohubba plugin were utilized for a thorough examination. Through this analysis, VCAM1 was identified as the core gene in the network, highlighting its crucial role in the immune-relevant genes associated with DN (**Figure 2**) (**Supplementary Table S** **6**).

### 3.3 Functional enrichment analysis

**Figure 3:**
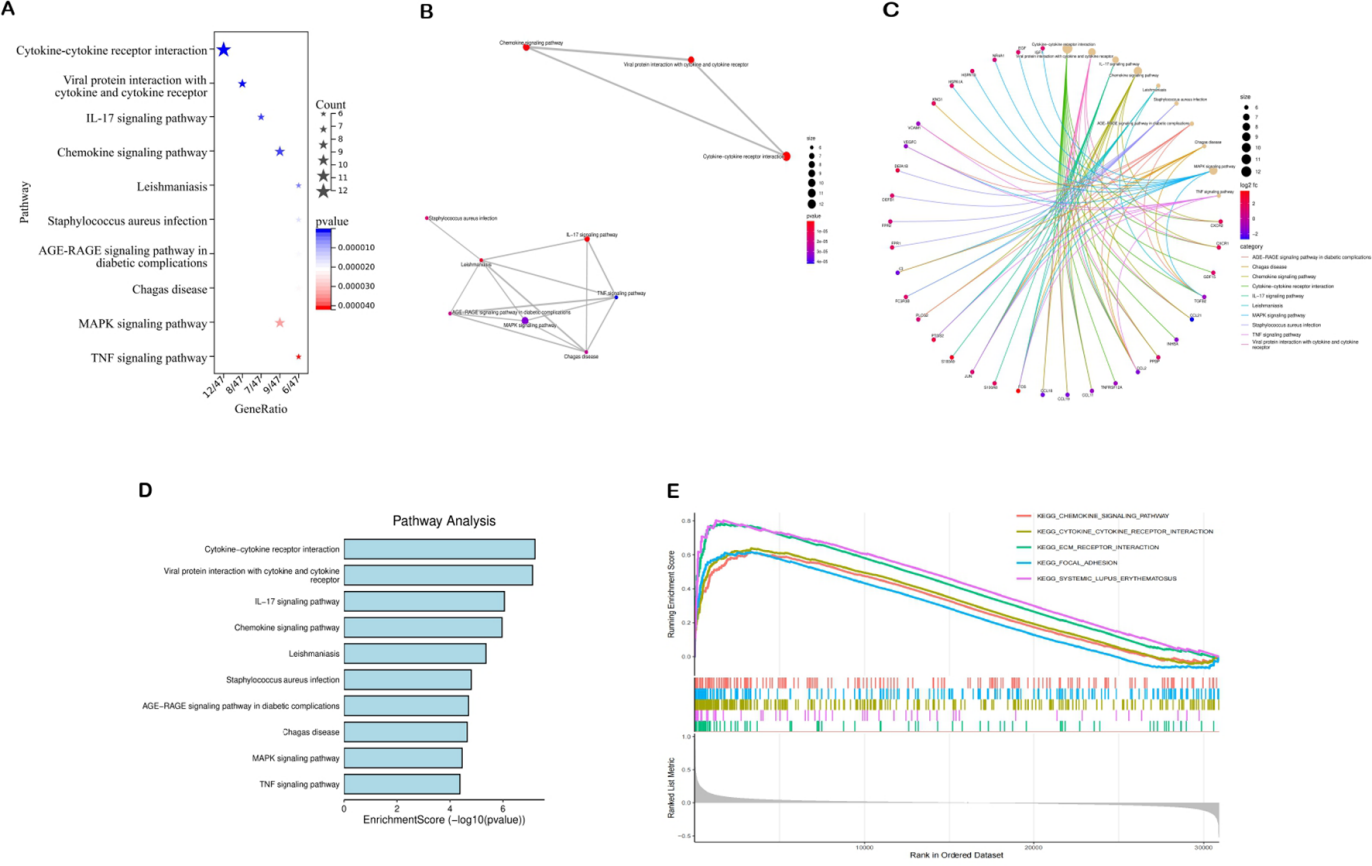
Results of pathway enrichment analysis. **(A–D)** Results of KEGG enrichment analysis. **(E)** Results of GSEA analysis.

The 74 common target genes were subjected to GO and KEGG enrichment analysis using R software. The GO enrichment analysis of biological processes (BP) indicated that DEGs are predominantly enriched in receptor-mediated endocytosis, humoral immune response, leukocyte chemotaxis, and cell chemotaxis (**Figure 2D**). In terms of cellular components (CC), the critical intersection targets were primarily enriched in immunoglobulin complex, blood microparticle, secretory granule lumen, and cytoplasmic vesicle lumen (**Figure 2E**). The enriched molecular function (MF) terms included receptor ligand activity, signaling receptor activator activity, antigen binding, and G protein-coupled receptor binding, among others (**Figure 2F**). The top 10 differentially expressed pathways related to DN were enriched in Cytokine-cytokine receptor interaction, IL-17 signaling pathway, Chemokine signaling pathway, AGE-RAGE signaling pathway in diabetic complications, MAPK signaling pathway, and TNF signaling pathway, as illustrated in **Figures 3A–D** (**Supplementary Table S** **7**).

### 3.4 The result of single gene GSEA analysis

We analyzed potential signaling pathways associated with the core gene via GSEA. The results of enrichment analysis indicate significant correlations between VCAM1 and ECM receptor interactions (NES = 2.4862, *p* < 0.001), systemic lupus erythematosus (NES = 2.3773, *p* < 0.001), cytokine receptor interactions (NES = 2.3231, *p* < 0.001), focal adhesion (NES = 2.1908, *p* < 0.001), chemokine signaling pathways (NES = 2.1666, *p* < 0.001), and other signaling pathways (**Figure 3**). These results indicate that the core gene VCAM1 is associated with immune response.

### 3.5 Diagnostic value of biomarkers

**Figure 4:**
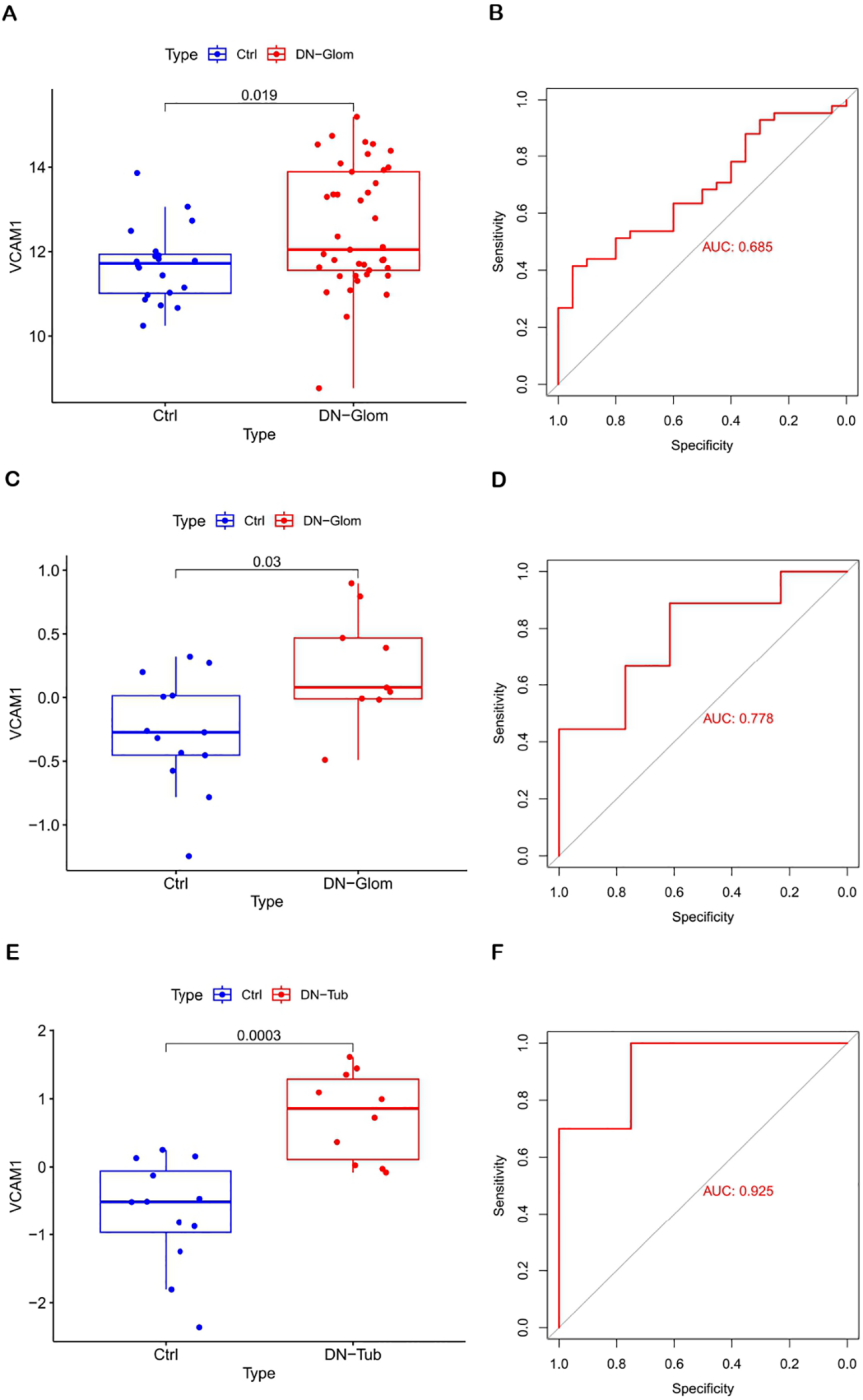
Diagnostic value of VCAM1. **(A, B)** Validation of DN characteristic the biomarker. Verification of expression levels in tissues and ROC curve analysis of VCAM1 from GSE96804. **(C, D)** Expression and ROC curve analysis of VCAM1 in tissues from GSE30528. **(E, F)** Expression and ROC curve analysis of VCAM1 in tissues from GSE30529.

The significance of the VCAM1 gene in DN was evaluated by analyzing its differential expression in three independent datasets: GSE96804 (test set), GSE30528 (validation set 1), and GSE30529 (validation set 2). The findings illustrated in **Figure 4** indicate that VCAM1 functions as a central immune gene with valuable diagnostic utility in discriminating between samples from individuals with DN and those from control subjects. Additionally, the levels of VCAM1 expression were notably elevated in DN tissues compared to control tissues. The AUC values derived from the ROC curves all exceeded 0.5, suggesting a high level of confidence in the use of VCAM1 as a diagnostic biomarker for DN.

### 3.6 Analysis of immune cell abundance

**Figure 5:**
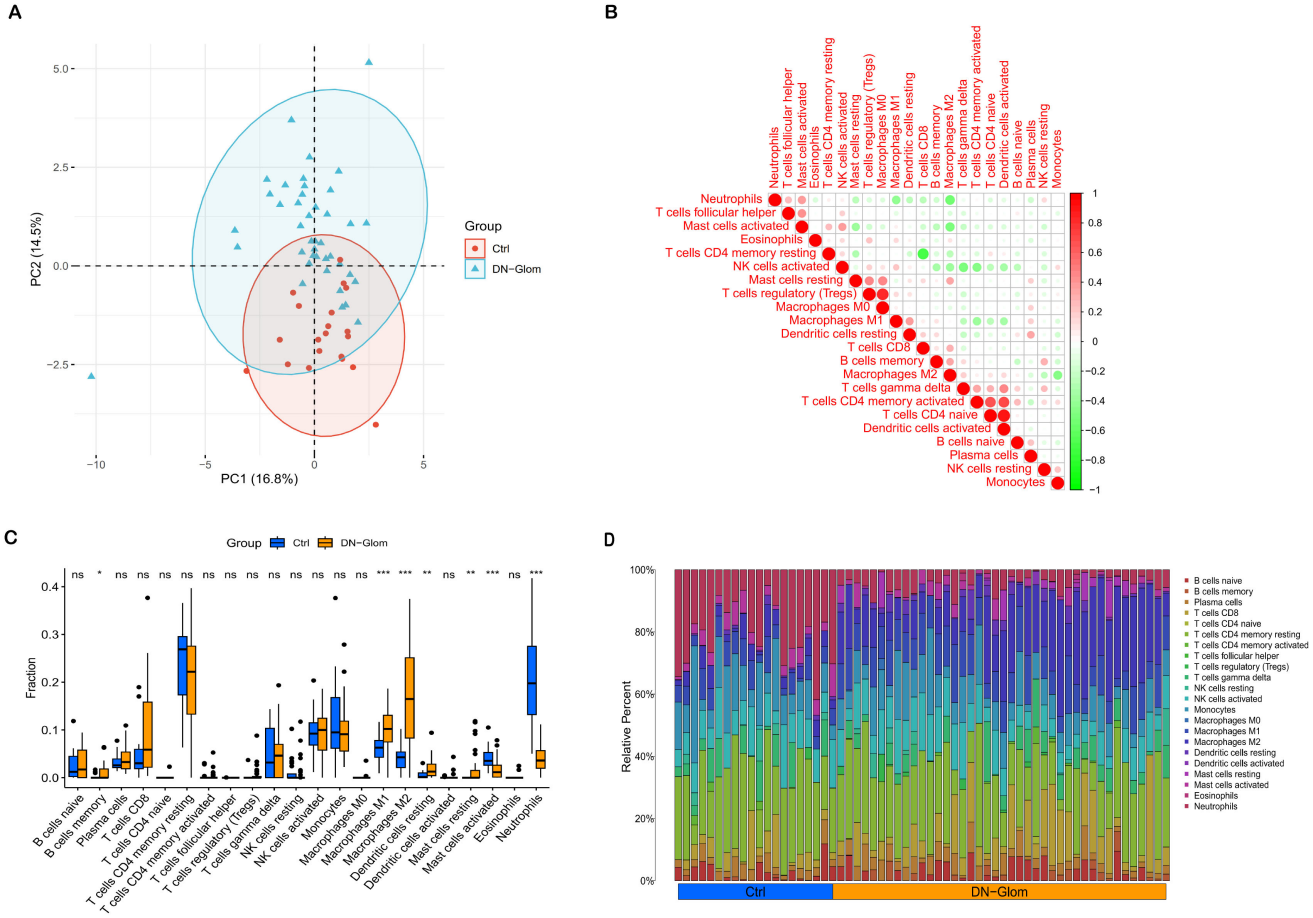
Immunological microenvironment analysis. **(A)** A PCA cluster plot reveals patterns of immune cell infiltration. This figure contrasts the immunological landscapes of the normal group (blue) and DN group (orange). **(B)** The correlation matrix for immune cells is depicted, with red indicating a positive correlation and green signifying a negative one. The intensity of color correlates with the strength of the relationship. **(C)** The comparative analysis highlights variances in immune infiltration between DN patients and control subjects, using blue to denote normal and yellow for DN renal tissue samples. **(D)** This section details relative proportions across 22 subpopulations of immune cells; statistical significance is denoted by asterisks. *P < 0.05 indicates significance, **P < 0.01 denotes high significance, and ***P < 0.001 signifies very high significance.

An examination of immune cell composition in DN kidney tissue revealed marked differences compared to healthy controls. Principal component analysis showed significant variation in immune cell infiltration, as depicted in **Figure 5**, suggesting these cells’ pivotal role in DN progression. Analysis of the GSE96804 dataset via CIBERSORTx, shown in **Figures 5C, D** (*P* < 0.05), indicated that monocytes, naïve B cells, memory B cells, plasma cells, CD8 T cells, activated NK cells and both types of macrophages (M1 and M2) were more prevalent in DN patients than controls. Furthermore, the study identified correlations among the proportions of 22 immune cell types within glomerular tissue (**Figure 5B**). The immune response involved in DN is complex and tightly regulated.

**Figure 6:**
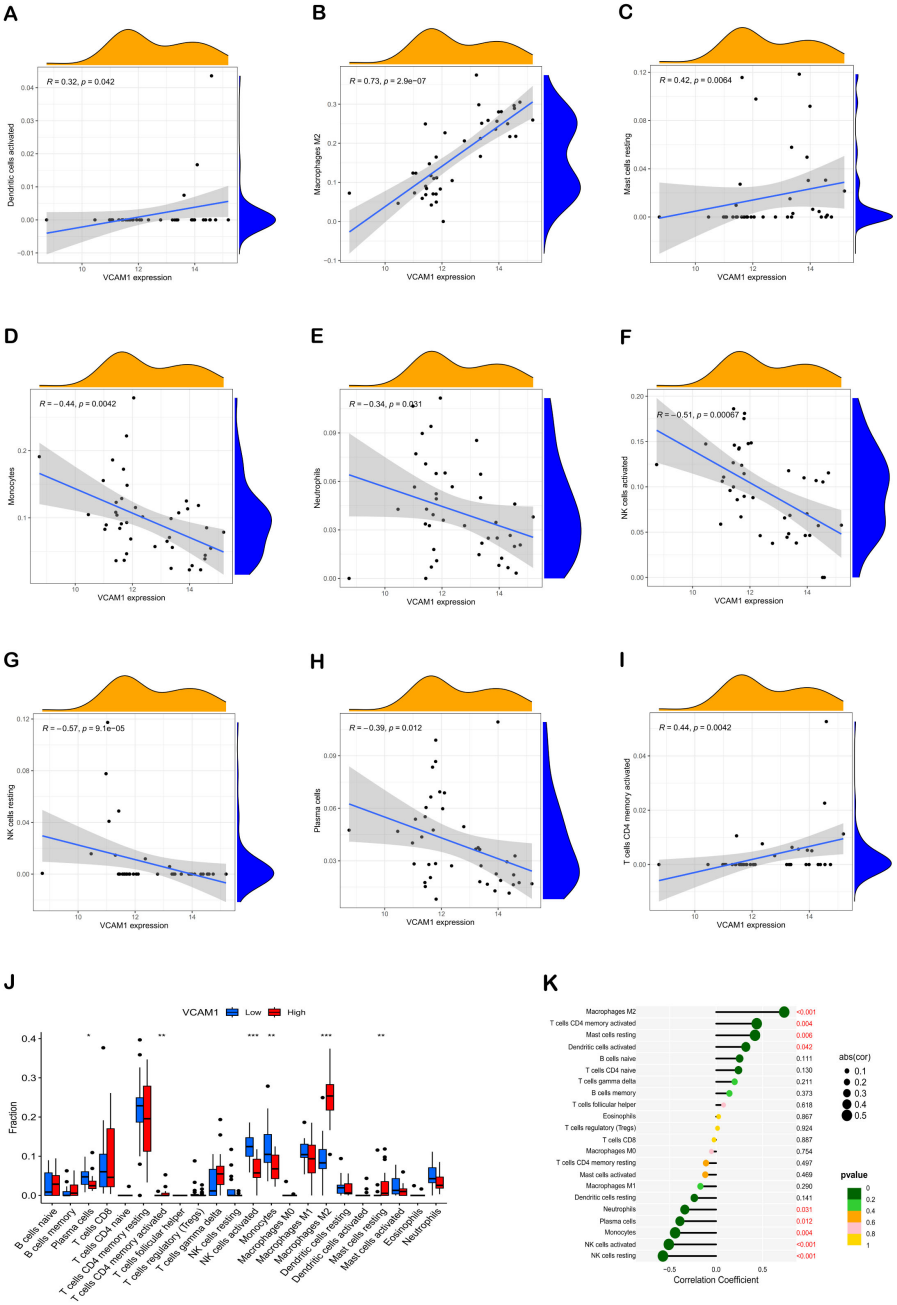
Correlation of hub genes with immune cells. Correlation analysis of VCAM1 expression with Dendritic cells activated, Macrophages M2, Mast cells resting, Monocytes, Neutrophils, NK cells activated, NK cells resting, Plasma cells, and T cells CD4 memory activated, respectively **(A–I)**. Comparative box plots depict the distribution of 22 immune cell types in subgroups characterized by high versus low VCAM1 expression levels **(J)**. The relationship between VCAM1 expression and invasive immune cells is illustrated. In comparison to the Low group, *P < 0.05, **P < 0.01, ***P < 0.001 **(K)**. Dot size indicates the correlation strength between central immune-related genes and various immune cells; larger dots signify stronger correlations, while smaller ones denote weaker associations. Additionally, the dot color reflects statistical significance: greener hues represent lower P values (suggesting higher significance), whereas more yellow tones indicate higher P values (implying lesser significance).

Furthermore, the Spearman correlation analysis indicated statistically significant positive associations between VCAM1 expression and Dendritic cells activated (r = 0.32, p = 0.042), Macrophages M2 (r = 0.73, p = 2.9e-07), Mast cells resting (r = 0.42, p = 0.0064), and T cells CD4 memory activated (r = 0.44, p = 0.0042). Conversely, significant negative correlations were observed between VCAM1 expression and Monocytes (r = -0.44, p = 0.0042), Neutrophils (r = -0.34, p = 0.031), NK cells activated (r = -0.51, p = 0.00067), NK cells resting (r = 0.57, p = 9.1e-05), and Plasma cells (r = -0.39, p = 0.012) (**Figures 6A–I**).

### 3.7 Relationship between VCAM1 and the immune microenvironment in DN

Correlation analysis was conducted in this study to investigate the association between VCAM1 expression and the expression levels of characteristic genes in 22 types of immune infiltrating cells. The findings, depicted in **Figure 6**, demonstrated statistically significant negative correlations between VCAM1 and Plasma cells, NK cells resting, NK cells activated, Monocytes, and Neutrophils (*P* < 0.05) (**Figure 6J**). Conversely, VCAM1 exhibited significant positive correlations with T cells CD4 memory activated, Macrophages M2, Dendritic cells activated, and Mast cells resting (*P* < 0.05). These findings offer additional evidence supporting the impact of VCAM1 expression on the immunological activity of immune cells (**Figure 6K**).

### 3.8 General biochemical indicators of the kidney

**Figure 7:**
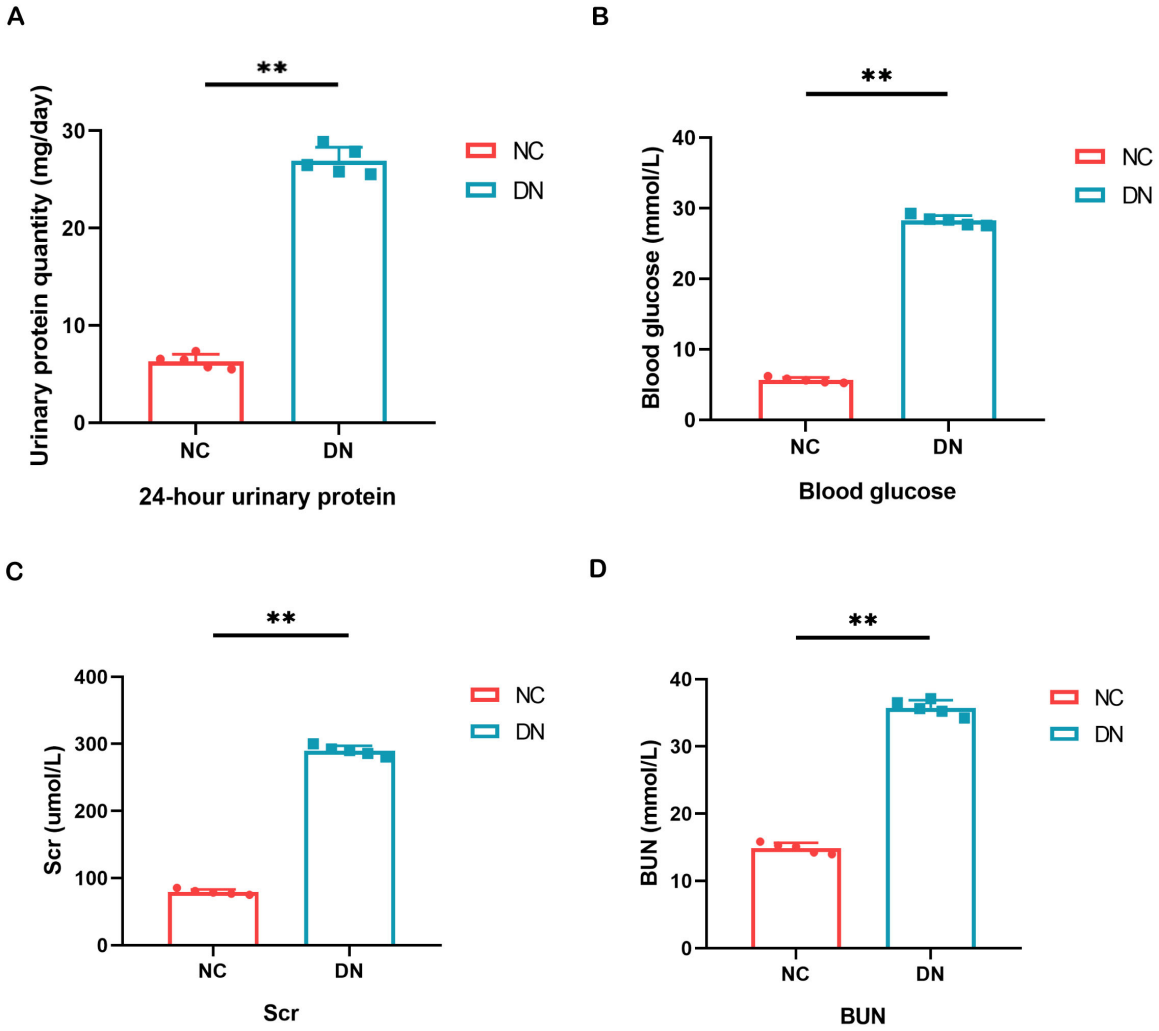
Result of general biochemical indices of the kidney. **(A)** Quantitative alterations in the 24-hour urine protein of rats. **(B)** Variations in the blood glucose levels of rats. **(C, D)** The serum BUN and Scr levels were assessed in each group of rats. In comparison to the NC group, **P < 0.01.

As a model of type 2 DN, we used STZ-induced SD rats to further validate the diagnostic value of the biomarker. The fasting blood glucose levels were consistently monitored and maintained above 16.7 mmol/L to induce DN in the mice. Subsequently, measurements of 24-hour urine protein quantification, blood glucose, BUN, and Scr were conducted, as these are essential clinical indicators for evaluating renal function. These findings demonstrated significantly elevated levels of 24-hour urine protein quantification, blood glucose, BUN, and Scr in the DN group compared to the normal control group (**Figure 7**).

### 3.9 Pathological changes of DN rats model

**Figure 8:**
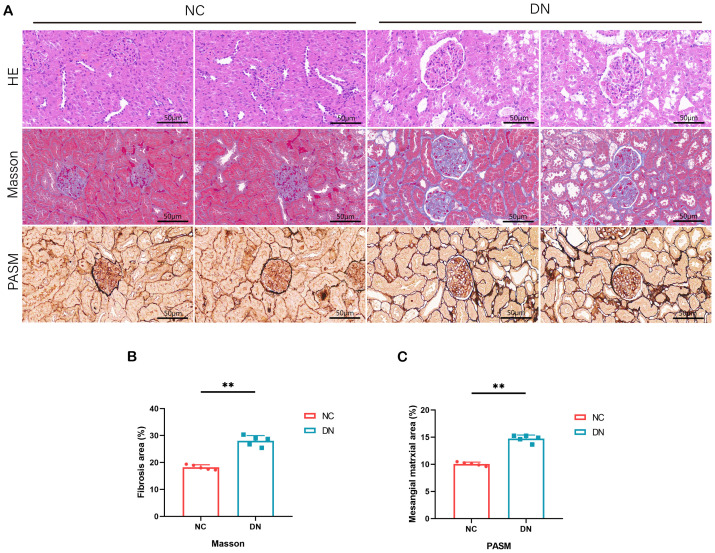
Pathological staining results in rats. **(A)** HE staining of renal tissue in DN rats (x 200, Scale bar = 50 µm). MASSON staining of renal tissue in DN rats (x 200, Scale bar = 50 µm). PASM staining of renal tissue in DN rats (x 200, Scale bar = 50 µm). **(B)** The area percentage of renal tissue fibrosis in each group of rats. In comparison to the NC group, **P < 0.01. **(C)** Area percentage of positive substances in each group of rats. In comparison to the NC group, **P < 0.01.

Microscopic examination of renal tissues stained with various histological techniques, such as HE, MASSON, and PAS, revealed thylakoid proliferation, enlarged thylakoid stroma, and irregular thickening of glomerular and tubular basement membranes in mice within the DN group. These findings confirm the successful establishment of a type 2 DN model in SD rats through the induction of high glucose, high lipid, and streptozotocin (**Figures 8A–C**).

### 3.10 Expression of VACM1 in renal tissue

**Figure 9:**
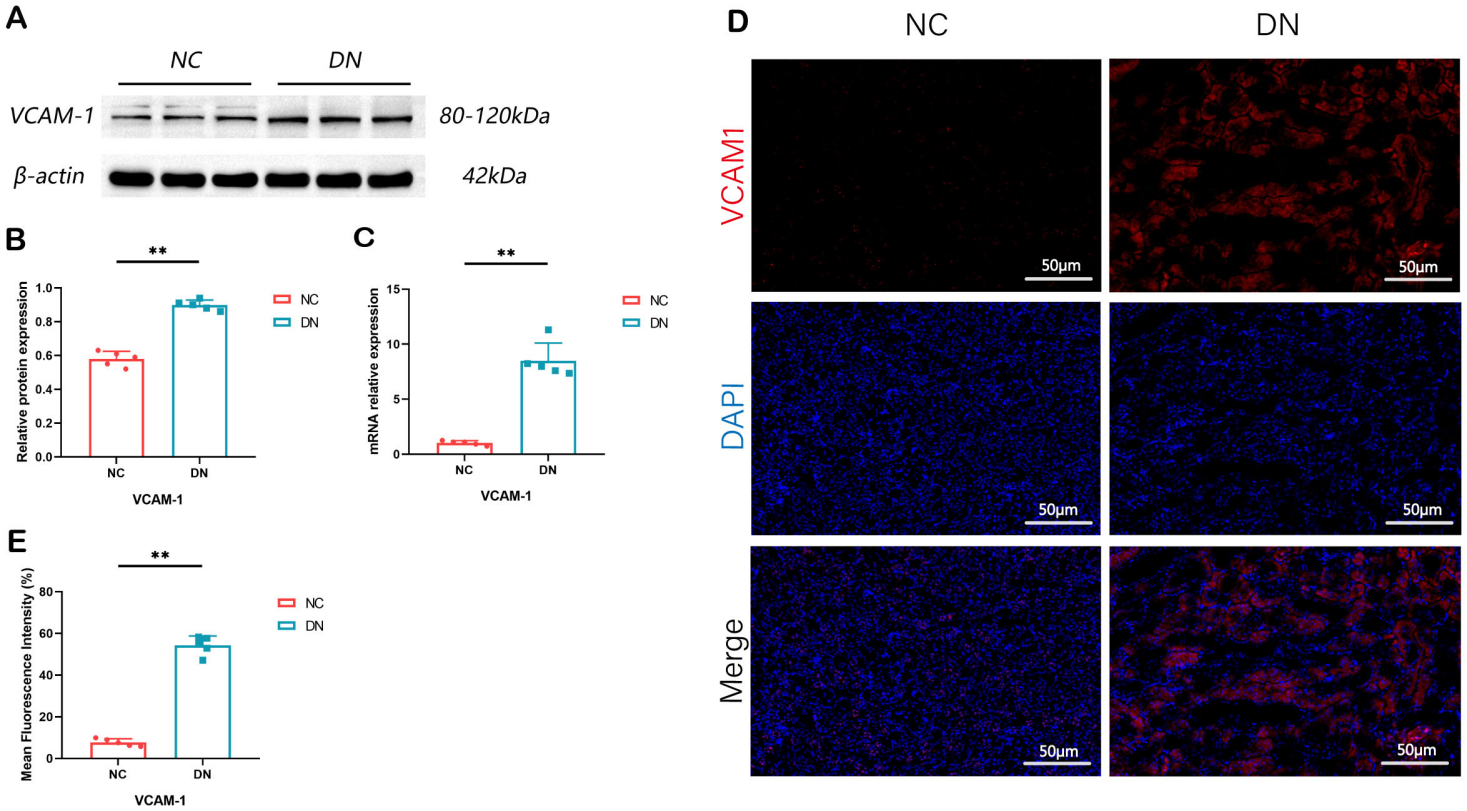
Expression of VCAM1 in rat model of DN. **(A)** The renal tissue of rats showed expression bands of VCAM1 proteins. **(B)** The protein levels of VCAM1 in the renal tissue of rats were measured and compared. In comparison to the NC group, **P < 0.01. **(C)** The relative expression levels of VCAM1 mRNA in the renal tissue of rats were assessed. The observed difference, when compared to the NC group, is **P < 0.01. **(D, E)** Immunofluorescence staining of VCAM1 in renal tissue of rats (x 200, Scale bar = 50 µm). Compared with the NC group, **P < 0.01. We presented the data as the mean + SD, while p < 0.05 indicates significance.

In order to confirm the findings, WB, RT qPCR, and IF assays were performed. The outcomes revealed a marked elevation in the expression levels of VACM1 protein, mRNA, and IF in the DN group. These findings suggest that the results obtained from data analysis are dependable and hold promise for further research endeavors. (*P < 0.05, **P < 0.01) (**Figure 9**).

## 4 Discussion

DN is a persistent inflammatory condition intricately linked to immune responses, resulting in podocyte damage, proteinuria, and diminished renal function (29). Immunomodulatory elements and the participation of immune cells are recognized as pivotal factors in the pathogenesis and advancement of DN (30). The principal aim of this research endeavor was to discern a novel immune prognostic indicator for DN, with the potential to enhance prognostic accuracy for DN patients and inform therapeutic strategies. Additionally, the study aimed to explore how immune cell penetration affects DN, providing valuable knowledge about the disease’s fundamental processes. Through the identification of distinct biomarkers and the analysis of immune cell infiltration patterns, this study aimed to enhance comprehension and treatment strategies for DN, potentially enhancing patient prognosis and treatment efficacy.

In the current investigation, a total of 74 DEGs were identified as significantly expressed in DN, with one hub biomarker also being identified. Additional investigation is required to establish the full extent of their role in the development of DN. Analysis of GO terms showed that IrDEGs were mainly enriched in processes linked to humoral immune response, immune response activation, leukocyte-mediated immunity, and complement activation through the classical pathway, potentially impacting the advancement of DN via interactions in the Cytokine-cytokine receptor pathway, IL-17 signaling pathway, Chemokine signaling pathway, AGE-RAGE signaling pathway in diabetic complications, MAPK signaling pathway, and TNF signaling pathway. Li, Bo et al. (30) demonstrated that the hub genes associated with DN patients are primarily enriched in processes related to humoral immune response, immune response activation, leukocyte-mediated immunity, and classical pathway complement activation, findings that align with our own research. Increasing evidence suggests that the complement system plays a role in the progression of DN. Levels of mannose-binding lectin, a molecule that recognizes patterns in the innate immune system, have been found to be a dependable indicator of the progression and worsening of this specific illness. Furthermore, studies suggest that MBL, H-ficolin, C3 complement component and the membrane attack complex could be involved in causing kidney damage in a high blood sugar setting (31–33). Additionally, the IL-17 and AGE-RAGE pathways have been found to play a crucial role in the progression of diabetic complications in those with DN. Th17 cells, a distinct subset of proinflammatory CD4+ T-effector cells, exhibit differential characteristics compared to Th1 and Th2 cell populations. Elevated levels of Th17 cytokines, including IL-17A, have been implicated in the stimulation of proinflammatory mediators and macrophage infiltration, exacerbating renal damage in DN (34). Since the initial discovery of RAGE, there has been an increasing amount of evidence indicating that AGEs/RAGE plays a significant role in the progression of DN (35, 36). Advanced glycation end products can attach to receptors for advanced glycation end products on the cell membrane, initiating intracellular signaling pathways such as AGEs-RAGE, NF-κB, and AGEs-RAGE-TGFβ1. The activation results in the release of different chemokines and growth factors, along with the multiplication of glomerular mesangial cells and initiation of podocyte apoptosis, ultimately worsening DN.

Our study’s results confirm the importance of VCAM1 as a key gene in the progression of DN, as shown by its expression and location. The finding emphasizes the possibility of VCAM1 being a favorable focus for treatment approaches and a useful indicator for detecting DN. VCAM1, a member of the Immunoglobulin superfamily, was discovered to be increased in infiltrating endothelial and renal tubulointerstitial cells in experimental DN. Moreover, the levels of its expression were correlated with the extent of proteinuria (37). The literature suggests that mesangial cells are essential for the structural and functional integrity of renal tubules, and their depletion can result in the deterioration of renal tubules (37). The results of the GSEA show that pathways associated with VCAM1 include interactions with the extracellular matrix (ECM), systemic lupus erythematosus, interactions with cytokines and cytokine receptors, focal adhesion, and signaling pathways for chemokines. Our results align with existing research indicating the significance of ECM receptor interactions in the development of DN (38). Li et al. identified COL6A3, COL1A2, THBS2, CD44, and FN1 as potential promoters of DN progression through the ECM-receptor interaction pathway, suggesting them as promising therapeutic targets (39). Additionally, cytokines and cytokine receptors have been implicated in macrophage/monocyte recruitment in animal models of DN and are associated with interstitial inflammation progression (40, 41).

Immune dysregulation is prevalent in DN and contributes to disease advancement (42). This study utilized the CIBERSORTx algorithm to assess immune cell infiltration, revealing increased enrichment of monocytes, naïve B cells, memory B cells, plasma cells, CD8 T cells, activated NK cells, and Macrophages M1 and Macrophages M2 in DN patients than in healthy controls. Bohle et al. (43) identified the presence of inflammatory cell infiltration through immunostaining in renal biopsy samples obtained from 488 individuals with DN. Inflammation, a multifaceted biological process crucial for combating microbial pathogens and facilitating tissue repair following injury, involves the pivotal participation of macrophages in modulating both innate and adaptive immune responses in DN (44, 45). Furthermore, emerging evidence suggests that B cells, neutrophils, and DCs accumulate in the glomeruli and interstitium even during the early stages of DN, exerting a significant regulatory influence on the pathogenesis of the disease (46, 47).

Significantly, VCAM1 has been demonstrated to be associated with immune cell infiltrations during the progression of DN. VCAM1 acts as a binding molecule on activated endothelial surfaces, aiding in the attachment and movement of white blood cells across epithelial barriers by interacting with white blood cell receptors, thus triggering an inflammatory reaction (48). The role of VCAM1 in mediating leukocyte adhesion to the endothelium is crucial. In an animal study utilizing the MRL/lpr murine model of lupus nephritis, increased expression of VCAM1 was observed in the endothelium, cortical tubules, and glomeruli of the kidneys (43). Patients diagnosed with type 2 diabetes exhibit notably increased serum concentrations of VCAM1, with a strong correlation between VCAM1 levels and the severity of albuminuria (49). These findings suggest a potential role for inflammation and immune response in the modulation of glomerular damage and highlight the diagnostic utility of VCAM1 in identifying diabetes-induced glomerular injury. Hence, it is hypothesized that VCAM1 may exhibit a strong association with immune infiltration in DN and possess significant diagnostic utility as a potential biomarker for DN in forthcoming diagnostic applications. Furthermore, the expression levels of VCAM1 were confirmed at the animal level through RT qPCR, WB, and IF techniques.

The objective of this research was to discover biomarkers for DN and explore the influence of immune cell infiltration on DN. Experimental verification was also conducted at the animal level. However, the study is limited by the validation of biomarkers only in DN rats without clinical data support and a limited number of samples. Hence, additional confirmation of the findings is required through experiments conducted in living organisms and in laboratory settings, along with real-world applications.

Previous research on DN and immune infiltration has been deficient in studies focusing exclusively on podocytes and has lacked sufficient experimental validation. Our study addresses this gap by innovatively identifying a pivotal gene in podocytes that is closely associated with DN and immune response, demonstrating high diagnostic efficacy. However, several limitations should be acknowledged. Firstly, the specific mechanisms underlying the role of the identified hub gene in DN require further investigation, which will be the primary focus of our subsequent research. Additionally, the expression levels and predictive performance of VCAM1 in other renal diseases commonly associated with DN, such as membranous nephropathy, need to be evaluated. Consequently, future research should focus on investigating the expression of VCAM1 in various chronic kidney diseases.

## 5 Conclusion

To sum up, this research employed thorough bioinformatics examination to identify differences in immune cell penetration among individuals with DN and those who are healthy. This study identified an immune-related biomarker associated with the advancement of DN, improving understanding of the role of immune-related genes in DN development and aiding in the creation of new diagnostic and treatment methods. Future validation studies with supplementary DN patient samples and models are warranted to explore the clinical application fully.

## Data Availability

The datasets presented in this study can be found in online repositories. The names of the repository/repositories and accession number(s) can be found in the article/**Supplementary material**.
