# Supplementary material for: VCAM1: an effective diagnostic marker related to immune cell infiltration in diabetic nephropathy
Source: Front Endocrinol (Lausanne). 2024 Sep 10;15:1426913. doi: 10.3389/fendo.2024.1426913 (PMC11420029; doi:10.3389/fendo.2024.1426913)
Supplement: Supplementary file 1 [file Table1.docx]

**Supplementary information**

**Table 1 Detailed information of datasets used in this study**

| **Dataset ID** | **Organism** | **Platform** | **normal samples vs. DN samples** | **Number of differential genes** |
| --- | --- | --- | --- | --- |
| GSE96804 | Human | GPL17586 | 20 vs. 41 (60) | 617 |

**Table S2 PCR Reaction System**

| **Reagent** | **volume/μL** |
| --- | --- |
| 2×SYBR qPCR Master Mix | 5 |
| ddcwater | 4.3 |
| cDNA sample | 0.3 |
| F | 0.2 |
| R | 0.2 |

**Table S3 PCR Reaction Procedure**

| **Loop Steps** | **Temperature（℃）** | **Time (seconds)** | **Number of cycles (times)** |
| --- | --- | --- | --- |
| 1 | 95 | 30 | 1 |
| 2 | 95 | 10 | 2-4 step cycle 40 |
| 3 | 60 | 30 |  |
| 4 | 72 | 10 |  |
| 5 | 95 | 15 | 1 |
| 6 | 60 | 60 | 1 |
| 7 | 95 | 15 | 1 |

**Table S4 Primer Information**

| **Primers** | | **Sequence** | **Amplified fragment size (bp)** |
| --- | --- | --- | --- |
| GAPDH | F | GATGACATCAAGAAGGTGGTGA | 192 |
|  | R | ACCCTGTTGCTGTAGCCATATTC |  |
| VCAM1 | F | GTGACGATGACATGTGCCA | 180 |
|  | R | TTTGTCTCTCCCAACCAGATT |  |

**Table S5 expression of DEGs in DN**

| **Name** | **P.Value** | **adj.P.Val** | **change** |
| --- | --- | --- | --- |
| FOS | 15.69450478 | 3.54E-23 | UP |
| DUSP1 | 15.15576471 | 1.96E-22 | UP |
| SNORD3D | 12.25649917 | 3.72E-18 | UP |
| CXCR2 | 11.9384061 | 1.17E-17 | UP |
| SNORD3B-1 | 11.89570785 | 1.37E-17 | UP |
| SNORD3B-2 | 11.89570785 | 1.37E-17 | UP |
| PDK4 | 11.88329611 | 1.43E-17 | UP |
| SNORD3C | 11.8811825 | 1.44E-17 | UP |
| HIST2H2AA4 | 11.67318668 | 3.07E-17 | UP |
| HSD17B14 | 11.56477601 | 4.57E-17 | UP |
| SNORD3A | 11.35907735 | 9.74E-17 | UP |
| HSPA1A | 11.19976927 | 1.76E-16 | UP |
| HSPA1B | 10.55100218 | 2.00E-15 | UP |
| DANCR | 10.52562539 | 2.20E-15 | UP |
| CXCR1 | 10.50309217 | 2.40E-15 | UP |
| MORN2 | 10.47052651 | 2.71E-15 | UP |
| NEBL-AS1 | 10.34308855 | 4.40E-15 | UP |
| BLVRB | 10.33389023 | 4.56E-15 | UP |
| ZFP36 | 10.31629366 | 4.88E-15 | UP |
| G6PC | 10.28455888 | 5.50E-15 | UP |
| HIST1H2BD | 9.908711094 | 2.33E-14 | UP |
| CKB | 9.878718278 | 2.61E-14 | UP |
| S100A8 | 9.458884584 | 1.33E-13 | UP |
| PCBD1 | 9.392146409 | 1.72E-13 | UP |
| JUN | 9.369924972 | 1.88E-13 | UP |
| ECH1 | 9.337258018 | 2.13E-13 | UP |
| SNORD66 | 9.177353953 | 3.99E-13 | UP |
| NFIL3 | 9.007842877 | 7.75E-13 | UP |
| SAT2 | 8.735413278 | 2.27E-12 | UP |
| S100A12 | 8.727283049 | 2.34E-12 | UP |
| S100A9 | 8.647016484 | 3.21E-12 | UP |
| KIAA1191 | 8.623174935 | 3.53E-12 | UP |
| MED11 | 8.469654735 | 6.48E-12 | UP |
| VNN2 | 8.423899879 | 7.77E-12 | UP |
| GSTA3 | 8.405612128 | 8.35E-12 | UP |
| AKR7A3 | 8.362103326 | 9.92E-12 | UP |
| ERRFI1 | 8.324338243 | 1.15E-11 | UP |
| CDH10 | 8.323376141 | 1.16E-11 | UP |
| PTGS2 | 8.225388381 | 1.71E-11 | UP |
| JUNB | 8.075799954 | 3.09E-11 | UP |
| BTG2 | 8.073204581 | 3.12E-11 | UP |
| HBA1 | 8.065710853 | 3.22E-11 | UP |
| LOC727944 | 8.054297152 | 3.37E-11 | UP |
| CYP27B1 | 7.930140375 | 5.52E-11 | UP |
| CA2 | 7.855096967 | 7.44E-11 | UP |
| FCGR3B | 7.839253423 | 7.92E-11 | UP |
| ETFB | 7.778801984 | 1.01E-10 | UP |
| MAOA | 7.719078041 | 1.28E-10 | UP |
| RNU5B-1 | 7.69592903 | 1.40E-10 | UP |
| GDF15 | 7.678299665 | 1.50E-10 | UP |
| HIST1H1E | 7.564238885 | 2.37E-10 | UP |
| A1CF | 7.489579615 | 3.19E-10 | UP |
| TREM1 | 7.488174428 | 3.21E-10 | UP |
| HBB | 7.470619733 | 3.44E-10 | UP |
| SMCO3 | 7.46397004 | 3.53E-10 | UP |
| FPR1 | 7.410700412 | 4.37E-10 | UP |
| DCXR | 7.39572756 | 4.63E-10 | UP |
| ANXA9 | 7.348965296 | 5.58E-10 | UP |
| AQP9 | 7.308362782 | 6.56E-10 | UP |
| SNORD105B | 7.210946147 | 9.67E-10 | UP |
| FPR2 | 7.093505728 | 1.54E-09 | UP |
| HES1 | 7.038060968 | 1.92E-09 | UP |
| ALPL | 7.023341198 | 2.04E-09 | UP |
| ACSM5 | 6.995911555 | 2.27E-09 | UP |
| ALDH2 | 6.920828325 | 3.06E-09 | UP |
| NR4A1 | 6.904284213 | 3.27E-09 | UP |
| CYP2B6 | 6.878356008 | 3.62E-09 | UP |
| HIST2H2BE | 6.805473118 | 4.83E-09 | UP |
| CRYL1 | 6.725612993 | 6.63E-09 | UP |
| TMEM150C | 6.720200573 | 6.77E-09 | UP |
| NAP1L2 | 6.678848837 | 7.98E-09 | UP |
| LPL | 6.673753912 | 8.14E-09 | UP |
| ANKS4B | 6.664811013 | 8.43E-09 | UP |
| ESM1 | 6.658832496 | 8.63E-09 | UP |
| CYB5A | 6.630158634 | 9.67E-09 | UP |
| GSTA5 | 6.61505605 | 1.03E-08 | UP |
| ECHS1 | 6.598657706 | 1.09E-08 | UP |
| AK4P3 | 6.581182674 | 1.17E-08 | UP |
| HBA2 | 6.564985075 | 1.25E-08 | UP |
| FAM180A | 6.538634006 | 1.39E-08 | UP |
| FXYD1 | 6.533245783 | 1.42E-08 | UP |
| C9orf66 | 6.504240491 | 1.59E-08 | UP |
| TPPP3 | 6.501953034 | 1.60E-08 | UP |
| SNORA22 | 6.476180493 | 1.77E-08 | UP |
| PTGDS | 6.473262911 | 1.79E-08 | UP |
| G0S2 | 6.430231475 | 2.13E-08 | UP |
| C14orf164 | 6.332682923 | 3.12E-08 | UP |
| ACMSD | 6.330847374 | 3.14E-08 | UP |
| ALB | 6.296305658 | 3.59E-08 | UP |
| SNORA47 | 6.294459904 | 3.62E-08 | UP |
| CMBL | 6.257488907 | 4.18E-08 | UP |
| ALDH4A1 | 6.234786606 | 4.57E-08 | UP |
| SLC7A7 | 6.232805353 | 4.61E-08 | UP |
| VASN | 6.160862929 | 6.10E-08 | UP |
| PEPD | 6.135199004 | 6.75E-08 | UP |
| FBP1 | 6.126073667 | 6.99E-08 | UP |
| EGR1 | 6.123881968 | 7.05E-08 | UP |
| SMIM2-AS1 | 6.122710829 | 7.08E-08 | UP |
| LOX | 6.095942132 | 7.86E-08 | UP |
| BRE-AS1 | 6.082872577 | 8.27E-08 | UP |
| HPD | 6.079906046 | 8.37E-08 | UP |
| CTH | 6.061443175 | 8.99E-08 | UP |
| GMNC | 6.007684648 | 1.11E-07 | UP |
| PDZK1IP1 | 6.007618509 | 1.11E-07 | UP |
| RGS2 | 5.90728256 | 1.64E-07 | UP |
| OTTHUMG00000160692 | 5.901137459 | 1.67E-07 | UP |
| PI3 | 5.895051788 | 1.71E-07 | UP |
| PRODH2 | 5.878749072 | 1.83E-07 | UP |
| ACY3 | 5.854631843 | 2.00E-07 | UP |
| CRYAA | 5.846870822 | 2.06E-07 | UP |
| EGF | 5.797378846 | 2.50E-07 | UP |
| PCK1 | 5.792601128 | 2.55E-07 | UP |
| RNF152 | 5.77966904 | 2.68E-07 | UP |
| OTTHUMG00000036478 | 5.77441462 | 2.73E-07 | UP |
| FRY-AS1 | 5.762811857 | 2.85E-07 | UP |
| OTTHUMG00000162500 | 5.761411687 | 2.87E-07 | UP |
| USH1C | 5.758574285 | 2.90E-07 | UP |
| SNORA12 | 5.75726886 | 2.92E-07 | UP |
| NPL | 5.736493275 | 3.16E-07 | UP |
| AK4 | 5.733161982 | 3.20E-07 | UP |
| ASPA | 5.724434491 | 3.31E-07 | UP |
| GSTA2 | 5.71278627 | 3.46E-07 | UP |
| EPHX2 | 5.699497987 | 3.64E-07 | UP |
| CLEC4E | 5.676425531 | 3.98E-07 | UP |
| RASD1 | 5.648434399 | 4.43E-07 | UP |
| CTXN3 | 5.621498465 | 4.91E-07 | UP |
| OTTHUMG00000009791 | 5.589581816 | 5.54E-07 | UP |
| GIPC2 | 5.581023439 | 5.73E-07 | UP |
| PAIP2B | 5.572977237 | 5.91E-07 | UP |
| RNU5E-2P | 5.541630324 | 6.65E-07 | UP |
| LOC100996266 | 5.520312049 | 7.22E-07 | UP |
| ACY1 | 5.504126427 | 7.67E-07 | UP |
| SORD | 5.500347898 | 7.78E-07 | UP |
| ALDH6A1 | 5.484822708 | 8.26E-07 | UP |
| SELL | 5.460072168 | 9.07E-07 | UP |
| RNU4-2 | 5.44512707 | 9.60E-07 | UP |
| FABP1 | 5.4269579 | 1.03E-06 | UP |
| DPP6 | 5.388357129 | 1.19E-06 | UP |
| NAPSA | 5.360451995 | 1.32E-06 | UP |
| LINC00948 | 5.356808163 | 1.34E-06 | UP |
| DPEP1 | 5.344953076 | 1.40E-06 | UP |
| PRKAR2B | 5.335564323 | 1.45E-06 | UP |
| CETP | 5.264310909 | 1.90E-06 | UP |
| LOC100505664 | 5.242413102 | 2.06E-06 | UP |
| OTTHUMG00000156955 | 5.24185434 | 2.06E-06 | UP |
| RNU5D-2P | 5.240349716 | 2.08E-06 | UP |
| OTTHUMG00000002710 | 5.229808005 | 2.16E-06 | UP |
| HRSP12 | 5.229037338 | 2.16E-06 | UP |
| KHK | 5.217738115 | 2.26E-06 | UP |
| FMN2 | 5.206403187 | 2.36E-06 | UP |
| SNORA48 | 5.179003841 | 2.61E-06 | UP |
| L3MBTL3 | 5.170546417 | 2.69E-06 | UP |
| AQP7P3 | 5.133391275 | 3.09E-06 | UP |
| ABAT | 5.11109295 | 3.36E-06 | UP |
| OTTHUMG00000132691 | 5.072697613 | 3.87E-06 | UP |
| PBLD | 5.071234032 | 3.89E-06 | UP |
| SNORD14D | 5.059282206 | 4.07E-06 | UP |
| MIOX | 5.053017705 | 4.17E-06 | UP |
| LOC728290 | 5.05188838 | 4.18E-06 | UP |
| OTTHUMG00000036063 | 5.04807594 | 4.24E-06 | UP |
| MRO | 5.036659747 | 4.43E-06 | UP |
| OTTHUMG00000016695 | 5.001219663 | 5.04E-06 | UP |
| SNORD41 | 4.976410107 | 5.53E-06 | UP |
| APOH | 4.964131253 | 5.78E-06 | UP |
| PVALB | 4.949802412 | 6.09E-06 | UP |
| FOLH1B | 4.941855788 | 6.27E-06 | UP |
| GSTA1 | 4.941828617 | 6.27E-06 | UP |
| EGOT | 4.929257996 | 6.57E-06 | UP |
| APLN | 4.92122771 | 6.77E-06 | UP |
| SLC12A3 | 4.918873589 | 6.83E-06 | UP |
| CTSL2 | 4.917398993 | 6.86E-06 | UP |
| TNNI1 | 4.908031067 | 7.10E-06 | UP |
| SLC7A8 | 4.887789591 | 7.65E-06 | UP |
| EHHADH | 4.886914146 | 7.67E-06 | UP |
| NR4A2 | 4.879090518 | 7.89E-06 | UP |
| FABP3 | 4.874496338 | 8.03E-06 | UP |
| TM4SF5 | 4.861878004 | 8.41E-06 | UP |
| ARHGAP19 | 4.860787112 | 8.44E-06 | UP |
| OTTHUMG00000041417 | 4.79443731 | 1.07E-05 | UP |
| HSD17B2 | 4.794197149 | 1.08E-05 | UP |
| GLYATL1 | 4.767614064 | 1.18E-05 | UP |
| LRRC2 | 4.75064968 | 1.26E-05 | UP |
| FUT6 | 4.742751226 | 1.30E-05 | UP |
| PCOLCE2 | 4.71805868 | 1.42E-05 | UP |
| PTH1R | 4.705388785 | 1.48E-05 | UP |
| RHCG | 4.688150044 | 1.58E-05 | UP |
| GBA3 | 4.67991359 | 1.62E-05 | UP |
| AGMAT | 4.673949516 | 1.66E-05 | UP |
| NPHS1 | 4.64508071 | 1.84E-05 | UP |
| WDR72 | 4.617701839 | 2.03E-05 | UP |
| ABCC2 | 4.60688405 | 2.11E-05 | UP |
| HAO2 | 4.565700051 | 2.44E-05 | UP |
| CPXM1 | 4.553715948 | 2.55E-05 | UP |
| PPBP | 4.54625005 | 2.62E-05 | UP |
| SLC6A13 | 4.525961516 | 2.81E-05 | UP |
| GLYAT | 4.484800364 | 3.25E-05 | UP |
| PIPOX | 4.479933972 | 3.31E-05 | UP |
| SLC36A2 | 4.478965852 | 3.32E-05 | UP |
| LOC100505985 | 4.474159754 | 3.38E-05 | UP |
| DDC | 4.469651228 | 3.43E-05 | UP |
| SLC6A19 | 4.46543618 | 3.48E-05 | UP |
| RBP5 | 4.4586002 | 3.57E-05 | UP |
| ADH6 | 4.454832765 | 3.62E-05 | UP |
| DPYS | 4.443424623 | 3.76E-05 | UP |
| AFM | 4.442219833 | 3.78E-05 | UP |
| LOC101060277 | 4.413653383 | 4.18E-05 | UP |
| USP2 | 4.412815167 | 4.19E-05 | UP |
| EHD3 | 4.384806343 | 4.62E-05 | UP |
| WDR49 | 4.354285026 | 5.14E-05 | UP |
| OTTHUMG00000040386 | 4.350845376 | 5.20E-05 | UP |
| FAM151A | 4.345295183 | 5.31E-05 | UP |
| LINC00052 | 4.33083584 | 5.58E-05 | UP |
| SNORD14E | 4.29630365 | 6.29E-05 | UP |
| HGD | 4.295207 | 6.31E-05 | UP |
| TCF21 | 4.260082395 | 7.13E-05 | UP |
| PAH | 4.254555668 | 7.27E-05 | UP |
| ANGPTL3 | 4.218962233 | 8.21E-05 | UP |
| ALDOB | 4.204325223 | 8.64E-05 | UP |
| OTTHUMG00000014129 | 4.144517296 | 0.000105952 | UP |
| PLGLA | 4.111080419 | 0.000118707 | UP |
| HPGD | 4.106223432 | 0.000120679 | UP |
| DAO | 4.100393188 | 0.000123088 | UP |
| SNORD59B | 4.079140028 | 0.000132269 | UP |
| ACADSB | 4.064851892 | 0.000138808 | UP |
| CR1 | 4.02562458 | 0.000158407 | UP |
| APOM | 3.986370465 | 0.000180676 | UP |
| MT1H | 3.982786384 | 0.000182853 | UP |
| MT1G | 3.96170387 | 0.00019618 | UP |
| PLG | 3.949775032 | 0.00020413 | UP |
| MNDA | 3.925259063 | 0.000221453 | UP |
| OTTHUMG00000015137 | 3.90618658 | 0.000235898 | UP |
| CYP4A22 | 3.897123067 | 0.000243077 | UP |
| XPNPEP2 | 3.879590654 | 0.000257562 | UP |
| SLC2A2 | 3.869247815 | 0.000266492 | UP |
| NAT8B | 3.868351951 | 0.00026728 | UP |
| TYRO3 | 3.841776618 | 0.000291679 | UP |
| DEFB1 | 3.808075167 | 0.000325705 | UP |
| PSAT1 | 3.800308604 | 0.000334069 | UP |
| KNG1 | 3.799679955 | 0.000334755 | UP |
| TMEM207 | 3.760055275 | 0.000380825 | UP |
| SLC13A3 | 3.727772963 | 0.000422787 | UP |
| CALB1 | 3.66352187 | 0.000519826 | UP |
| KCNJ15 | 3.656814043 | 0.000531103 | UP |
| SLC7A9 | 3.625823614 | 0.000586304 | UP |
| BHMT | 3.602840965 | 0.000630731 | UP |
| TMEM174 | 3.589531402 | 0.000657907 | UP |
| BHMT2 | 3.538496461 | 0.000772808 | UP |
| ACSM2A | 3.535753262 | 0.000779496 | UP |
| METTL7B | 3.517434076 | 0.000825588 | UP |
| CYP4A11 | 3.495767056 | 0.000883448 | UP |
| FOXQ1 | 3.471187897 | 0.000953742 | UP |
| ACSM2B | 3.458828987 | 0.000991062 | UP |
| OTTHUMG00000163648 | 3.367610012 | 0.001312532 | UP |
| NAT8 | 3.328409003 | 0.001479033 | UP |
| CLDN8 | 3.256739061 | 0.001836162 | UP |
| UGT2B7 | 3.20564107 | 0.002138764 | UP |
| SLC47A1 | 3.203810099 | 0.002150432 | UP |
| SST | 3.194647158 | 0.002209724 | UP |
| AGXT2 | 3.186849496 | 0.002261387 | UP |
| CHI3L1 | 3.181484889 | 0.002297586 | UP |
| SLC22A8 | 3.143695049 | 0.002568404 | UP |
| KLK1 | 3.117223626 | 0.002775617 | UP |
| TMEM52B | 3.066337993 | 0.003218562 | UP |
| HSD11B2 | 3.053612217 | 0.003339222 | UP |
| SLC4A4 | 3.0479864 | 0.003393897 | UP |
| UMOD | 3.030071508 | 0.003573618 | UP |
| FMO1 | 2.915383557 | 0.004950995 | UP |
| SLC5A12 | 2.853594789 | 0.005883059 | UP |
| SLC22A6 | 2.839888068 | 0.00611067 | UP |
| ATP6V1G3 | 2.835433443 | 0.006186374 | UP |
| OTTHUMG00000162001 | 2.801714478 | 0.006788077 | UP |
| C19orf77 | 2.749280509 | 0.007831484 | UP |
| SLC17A1 | 2.747458938 | 0.007870248 | UP |
| DEFA1B | 2.679699435 | 0.009443486 | UP |
| IGF1 | 2.614443823 | 0.011225189 | UP |
| SLC27A2 | 2.605756894 | 0.011484163 | UP |
| TMEM27 | 2.59594942 | 0.011783062 | UP |
| CUBN | 2.471052779 | 0.016257672 | UP |
| SLC34A1 | 2.457772808 | 0.016813917 | UP |
| PLCG2 | 2.431516477 | 0.017964314 | UP |
| MIR4521 | -15.53451556 | 5.87E-23 | DOWN |
| OTTHUMG00000018564 | -12.67936739 | 8.27E-19 | DOWN |
| MIR4256 | -12.57098427 | 1.21E-18 | DOWN |
| MIR548D1 | -11.78860637 | 2.02E-17 | DOWN |
| MIR1256 | -11.10778894 | 2.47E-16 | DOWN |
| LOC642799 | -11.10282432 | 2.52E-16 | DOWN |
| VTRNA1-3 | -11.07623957 | 2.78E-16 | DOWN |
| NPIPA3 | -11.0547454 | 3.01E-16 | DOWN |
| MIR548AD | -10.9328655 | 4.75E-16 | DOWN |
| NPIPB5 | -10.88782683 | 5.62E-16 | DOWN |
| MIR548D2 | -10.80942114 | 7.54E-16 | DOWN |
| PFN1P2 | -10.79849806 | 7.86E-16 | DOWN |
| NPIPB3 | -10.71216937 | 1.09E-15 | DOWN |
| NPIPB11 | -10.63548921 | 1.45E-15 | DOWN |
| OTTHUMG00000154698 | -10.62974739 | 1.48E-15 | DOWN |
| MIR548AI | -10.58289824 | 1.77E-15 | DOWN |
| MIR1254-1 | -10.57164684 | 1.85E-15 | DOWN |
| LOC440354 | -10.46941848 | 2.72E-15 | DOWN |
| LOC613037 | -10.42452748 | 3.23E-15 | DOWN |
| SLC7A5P2 | -10.42264203 | 3.25E-15 | DOWN |
| NPIPA5 | -10.40138395 | 3.53E-15 | DOWN |
| NPIPL3 | -10.39296509 | 3.64E-15 | DOWN |
| SMG1P1 | -10.37477576 | 3.90E-15 | DOWN |
| RNA5SP195 | -10.3256478 | 4.71E-15 | DOWN |
| LOC595101 | -10.31007455 | 4.99E-15 | DOWN |
| LOC100506060 | -10.24358863 | 6.44E-15 | DOWN |
| LOC100190986 | -10.22028976 | 7.03E-15 | DOWN |
| MIR548AJ2 | -10.11006628 | 1.07E-14 | DOWN |
| MIR548O2 | -10.0435306 | 1.38E-14 | DOWN |
| LOC101060449 | -10.03870255 | 1.41E-14 | DOWN |
| SPDYE3 | -9.927374147 | 2.16E-14 | DOWN |
| LOC728734 | -9.810139623 | 3.40E-14 | DOWN |
| MIR548AA2 | -9.706919444 | 5.07E-14 | DOWN |
| MIR1285-2 | -9.687760468 | 5.46E-14 | DOWN |
| SPDYE8P | -9.332510435 | 2.17E-13 | DOWN |
| MIR548A3 | -9.244379141 | 3.07E-13 | DOWN |
| RNU7-29P | -9.24215819 | 3.09E-13 | DOWN |
| RNA5SP312 | -9.086126458 | 5.70E-13 | DOWN |
| RNA5SP315 | -9.086126458 | 5.70E-13 | DOWN |
| RNA5SP313 | -9.086126458 | 5.70E-13 | DOWN |
| SPDYE1 | -8.935290978 | 1.03E-12 | DOWN |
| ANXA1 | -8.905789451 | 1.16E-12 | DOWN |
| RNU7-53P | -8.899873967 | 1.18E-12 | DOWN |
| MIR297 | -8.753067421 | 2.11E-12 | DOWN |
| LUST | -8.698307554 | 2.62E-12 | DOWN |
| SPDYE6 | -8.673297392 | 2.90E-12 | DOWN |
| RNA5SP311 | -8.669009869 | 2.94E-12 | DOWN |
| RNA5SP314 | -8.669009869 | 2.94E-12 | DOWN |
| RNA5SP317 | -8.669009869 | 2.94E-12 | DOWN |
| PRINS | -8.664079342 | 3.00E-12 | DOWN |
| DOCK9 | -8.64111063 | 3.29E-12 | DOWN |
| SPDYE2 | -8.632283585 | 3.40E-12 | DOWN |
| MIR548F1 | -8.590278543 | 4.02E-12 | DOWN |
| MIR548T | -8.563574861 | 4.47E-12 | DOWN |
| RNU6-38 | -8.530286109 | 5.10E-12 | DOWN |
| RNU7-47P | -8.521830432 | 5.27E-12 | DOWN |
| MIR103A2 | -8.486890327 | 6.05E-12 | DOWN |
| RNA5SP187 | -8.481688138 | 6.18E-12 | DOWN |
| RNU7-48P | -8.47642682 | 6.31E-12 | DOWN |
| RNU7-45P | -8.473716501 | 6.38E-12 | DOWN |
| RNU7-7P | -8.430578261 | 7.56E-12 | DOWN |
| LINC00342 | -8.389980269 | 8.88E-12 | DOWN |
| SPDYE5 | -8.348604823 | 1.05E-11 | DOWN |
| HNRNPU-AS1 | -8.3480332 | 1.05E-11 | DOWN |
| ANKRD36 | -8.332133539 | 1.12E-11 | DOWN |
| MIR548X | -8.31967226 | 1.17E-11 | DOWN |
| GUSBP3 | -8.318043475 | 1.18E-11 | DOWN |
| SNRK-AS1 | -8.266035188 | 1.45E-11 | DOWN |
| RNU6-8 | -8.221758926 | 1.73E-11 | DOWN |
| OTTHUMG00000162373 | -8.179499974 | 2.05E-11 | DOWN |
| OTTHUMG00000162476 | -8.17337964 | 2.10E-11 | DOWN |
| ANKRD36B | -8.166574975 | 2.16E-11 | DOWN |
| MIR548AC | -8.155497975 | 2.25E-11 | DOWN |
| SMA5 | -8.154136857 | 2.26E-11 | DOWN |
| LOC100506123 | -8.144932504 | 2.35E-11 | DOWN |
| MIR548H4 | -8.132154175 | 2.47E-11 | DOWN |
| MIR30A | -8.130786142 | 2.49E-11 | DOWN |
| KIRREL-IT1 | -8.081772352 | 3.02E-11 | DOWN |
| PCDHGA10 | -8.076532562 | 3.08E-11 | DOWN |
| MIR644A | -8.072466319 | 3.13E-11 | DOWN |
| RNU7-25P | -8.042458149 | 3.53E-11 | DOWN |
| RNA5SP310 | -8.026445826 | 3.76E-11 | DOWN |
| RNA5SP316 | -8.026445826 | 3.76E-11 | DOWN |
| ACAP2-IT1 | -8.022370752 | 3.82E-11 | DOWN |
| MIR103B2 | -8.007392643 | 4.06E-11 | DOWN |
| RNU7-35P | -7.980770606 | 4.51E-11 | DOWN |
| OTTHUMG00000162597 | -7.971539694 | 4.68E-11 | DOWN |
| EMP1 | -7.963800237 | 4.83E-11 | DOWN |
| MIR548H2 | -7.950903541 | 5.08E-11 | DOWN |
| RNU7-11P | -7.949184628 | 5.12E-11 | DOWN |
| MIR548H3 | -7.943508897 | 5.23E-11 | DOWN |
| GUSBP2 | -7.857689323 | 7.36E-11 | DOWN |
| RNU6-83P | -7.83247334 | 8.14E-11 | DOWN |
| OTTHUMG00000032185 | -7.827772526 | 8.29E-11 | DOWN |
| GUSBP9 | -7.798520202 | 9.32E-11 | DOWN |
| LOC100996862 | -7.792670981 | 9.54E-11 | DOWN |
| RNU6-3 | -7.729581226 | 1.23E-10 | DOWN |
| GOLGA8A | -7.722739043 | 1.26E-10 | DOWN |
| NAIP | -7.711847019 | 1.32E-10 | DOWN |
| OTTHUMG00000163252 | -7.664731068 | 1.59E-10 | DOWN |
| OTTHUMG00000059495 | -7.649226052 | 1.69E-10 | DOWN |
| OTTHUMG00000002408 | -7.641462646 | 1.74E-10 | DOWN |
| RNU7-24P | -7.634308927 | 1.79E-10 | DOWN |
| LOC100272216 | -7.611012824 | 1.97E-10 | DOWN |
| OTTHUMG00000151782 | -7.556538277 | 2.44E-10 | DOWN |
| AGAP6 | -7.546389462 | 2.54E-10 | DOWN |
| OCLM | -7.539621373 | 2.61E-10 | DOWN |
| MIR503 | -7.532701117 | 2.69E-10 | DOWN |
| MIR5047 | -7.501917874 | 3.04E-10 | DOWN |
| ATP13A3 | -7.497516129 | 3.09E-10 | DOWN |
| PER3 | -7.491296749 | 3.17E-10 | DOWN |
| RNA5SP229 | -7.482395696 | 3.28E-10 | DOWN |
| SLC6A6 | -7.473956284 | 3.39E-10 | DOWN |
| MIR450B | -7.435604331 | 3.95E-10 | DOWN |
| RNA5SP320 | -7.433233211 | 3.99E-10 | DOWN |
| RNU7-13P | -7.430579185 | 4.03E-10 | DOWN |
| MIR548W | -7.416520697 | 4.27E-10 | DOWN |
| AGAP7 | -7.390560588 | 4.73E-10 | DOWN |
| MIR548AN | -7.356693215 | 5.41E-10 | DOWN |
| RNA5SP166 | -7.353440561 | 5.48E-10 | DOWN |
| GUCY1A3 | -7.339270277 | 5.80E-10 | DOWN |
| OTTHUMG00000152506 | -7.338795317 | 5.81E-10 | DOWN |
| SMA4 | -7.305967015 | 6.62E-10 | DOWN |
| RPL23AP32 | -7.281809819 | 7.29E-10 | DOWN |
| RPL36AP33 | -7.221613281 | 9.26E-10 | DOWN |
| RNU6-80 | -7.163605963 | 1.17E-09 | DOWN |
| RNU6-43 | -7.147239797 | 1.25E-09 | DOWN |
| MIR548C | -7.140432636 | 1.28E-09 | DOWN |
| THRB-IT1 | -7.132055572 | 1.32E-09 | DOWN |
| FLJ45340 | -7.12387512 | 1.37E-09 | DOWN |
| GOLGA8B | -7.105713064 | 1.47E-09 | DOWN |
| RNU7-40P | -7.098940781 | 1.51E-09 | DOWN |
| CACNA1C-AS4 | -7.095386708 | 1.53E-09 | DOWN |
| RNU7-61P | -7.052049989 | 1.82E-09 | DOWN |
| OTTHUMG00000160464 | -7.039901819 | 1.91E-09 | DOWN |
| RNU7-62P | -6.992480741 | 2.30E-09 | DOWN |
| RNA5SP20 | -6.98241667 | 2.40E-09 | DOWN |
| FN1 | -6.966137594 | 2.56E-09 | DOWN |
| MIR3911 | -6.909915466 | 3.20E-09 | DOWN |
| LOC399753 | -6.90671625 | 3.24E-09 | DOWN |
| OTTHUMG00000036552 | -6.890388177 | 3.45E-09 | DOWN |
| SLIT3 | -6.880688415 | 3.59E-09 | DOWN |
| OTTHUMG00000002525 | -6.852522344 | 4.01E-09 | DOWN |
| RNU7-57P | -6.82612539 | 4.45E-09 | DOWN |
| OTTHUMG00000001211 | -6.812462843 | 4.70E-09 | DOWN |
| RNU6-21P | -6.811790941 | 4.71E-09 | DOWN |
| OTTHUMG00000020112 | -6.794297918 | 5.05E-09 | DOWN |
| LRP2BP | -6.790321264 | 5.13E-09 | DOWN |
| OTTHUMG00000164860 | -6.758525978 | 5.82E-09 | DOWN |
| TAS2R30 | -6.748657043 | 6.05E-09 | DOWN |
| GPR18 | -6.693482534 | 7.53E-09 | DOWN |
| MIR3671 | -6.671823499 | 8.20E-09 | DOWN |
| SNORA70G | -6.625771207 | 9.84E-09 | DOWN |
| MIR1299 | -6.624932953 | 9.87E-09 | DOWN |
| OTTHUMG00000020327 | -6.586667437 | 1.15E-08 | DOWN |
| RNU7-10P | -6.527340065 | 1.45E-08 | DOWN |
| RNA5SP343 | -6.518446824 | 1.50E-08 | DOWN |
| MIR612 | -6.497171824 | 1.63E-08 | DOWN |
| LTBP1 | -6.405094381 | 2.35E-08 | DOWN |
| ANGPT2 | -6.404118115 | 2.35E-08 | DOWN |
| RNU7-28P | -6.375041484 | 2.64E-08 | DOWN |
| RERG-IT1 | -6.315881992 | 3.33E-08 | DOWN |
| RNU6-60 | -6.300207664 | 3.54E-08 | DOWN |
| PLK2 | -6.281196248 | 3.81E-08 | DOWN |
| TPM1 | -6.275153822 | 3.90E-08 | DOWN |
| KLF7-IT1 | -6.265460188 | 4.06E-08 | DOWN |
| OTTHUMG00000160733 | -6.259006819 | 4.16E-08 | DOWN |
| SNORD7 | -6.252578419 | 4.27E-08 | DOWN |
| OTTHUMG00000157034 | -6.23632274 | 4.55E-08 | DOWN |
| ASPN | -6.223216956 | 4.78E-08 | DOWN |
| ECM1 | -6.200410518 | 5.23E-08 | DOWN |
| ZNF638-IT1 | -6.180518865 | 5.65E-08 | DOWN |
| MIR570 | -6.169051242 | 5.91E-08 | DOWN |
| OTTHUMG00000161212 | -6.139978657 | 6.62E-08 | DOWN |
| MAGI2-AS1 | -6.135795623 | 6.73E-08 | DOWN |
| LOC100303749 | -6.123288908 | 7.07E-08 | DOWN |
| RNU6-45P | -6.11903929 | 7.19E-08 | DOWN |
| MIR186 | -6.088159584 | 8.10E-08 | DOWN |
| VEGFC | -6.080660171 | 8.34E-08 | DOWN |
| MIR3120 | -6.073051986 | 8.59E-08 | DOWN |
| FBN1 | -6.047805688 | 9.48E-08 | DOWN |
| BCL6B | -6.043136247 | 9.66E-08 | DOWN |
| LOC100287497 | -6.037761402 | 9.86E-08 | DOWN |
| OTTHUMG00000015002 | -6.032545449 | 1.01E-07 | DOWN |
| OTTHUMG00000152418 | -5.969403566 | 1.29E-07 | DOWN |
| MIR548A2 | -5.960554744 | 1.33E-07 | DOWN |
| RNA5SP450 | -5.949255255 | 1.39E-07 | DOWN |
| ZBTB20-AS2 | -5.928700806 | 1.51E-07 | DOWN |
| ENAH | -5.928568734 | 1.51E-07 | DOWN |
| RNF138P1 | -5.904928073 | 1.65E-07 | DOWN |
| ADAMTS1 | -5.823469205 | 2.26E-07 | DOWN |
| MIR604 | -5.822600927 | 2.27E-07 | DOWN |
| MIR30E | -5.769550435 | 2.78E-07 | DOWN |
| LUM | -5.769431893 | 2.78E-07 | DOWN |
| SCARNA7 | -5.768448131 | 2.79E-07 | DOWN |
| CCND2 | -5.743868108 | 3.07E-07 | DOWN |
| TGFBI | -5.711608061 | 3.48E-07 | DOWN |
| RNA5SP82 | -5.669863983 | 4.08E-07 | DOWN |
| MIR421 | -5.664003974 | 4.17E-07 | DOWN |
| MIR548K | -5.662172768 | 4.20E-07 | DOWN |
| PCDH18 | -5.604136728 | 5.24E-07 | DOWN |
| MMP2 | -5.530838131 | 6.93E-07 | DOWN |
| COL15A1 | -5.526949008 | 7.04E-07 | DOWN |
| IGHJ1 | -5.519494963 | 7.24E-07 | DOWN |
| MIR95 | -5.405612826 | 1.11E-06 | DOWN |
| MIR3916 | -5.398091472 | 1.15E-06 | DOWN |
| RNU6-82P | -5.318479461 | 1.55E-06 | DOWN |
| OTTHUMG00000019064 | -5.261901699 | 1.91E-06 | DOWN |
| ADAMTS5 | -5.25073399 | 2.00E-06 | DOWN |
| SYT11 | -5.243778486 | 2.05E-06 | DOWN |
| NT5E | -5.214342999 | 2.29E-06 | DOWN |
| MIR4263 | -5.111692668 | 3.35E-06 | DOWN |
| IGFBP6 | -5.086842248 | 3.68E-06 | DOWN |
| FABP5P1 | -5.072989671 | 3.87E-06 | DOWN |
| NID2 | -5.071520621 | 3.89E-06 | DOWN |
| SFRP2 | -5.071075559 | 3.90E-06 | DOWN |
| RNA5SP160 | -5.050335101 | 4.21E-06 | DOWN |
| PDE1A | -5.042223691 | 4.34E-06 | DOWN |
| SLC12A2 | -5.03969849 | 4.38E-06 | DOWN |
| SNORD75 | -5.027117669 | 4.58E-06 | DOWN |
| COL1A2 | -5.012193175 | 4.84E-06 | DOWN |
| RASSF9 | -5.00237828 | 5.02E-06 | DOWN |
| TGFB2 | -4.968791523 | 5.68E-06 | DOWN |
| SNORD63 | -4.963526856 | 5.79E-06 | DOWN |
| OTTHUMG00000151895 | -4.950143829 | 6.09E-06 | DOWN |
| MOXD1 | -4.933659218 | 6.47E-06 | DOWN |
| CCL21 | -4.926458232 | 6.64E-06 | DOWN |
| FABP5P7 | -4.92617396 | 6.65E-06 | DOWN |
| LOC645638 | -4.888719349 | 7.62E-06 | DOWN |
| RNA5SP268 | -4.83731735 | 9.19E-06 | DOWN |
| RNY3P6 | -4.835441683 | 9.25E-06 | DOWN |
| MIR3975 | -4.826860671 | 9.55E-06 | DOWN |
| OTTHUMG00000152518 | -4.815423021 | 9.95E-06 | DOWN |
| MARCKS | -4.783207353 | 1.12E-05 | DOWN |
| F2RL2 | -4.766674722 | 1.19E-05 | DOWN |
| AEBP1 | -4.764727052 | 1.20E-05 | DOWN |
| SNORA45 | -4.743111455 | 1.29E-05 | DOWN |
| IGHG4 | -4.724300239 | 1.38E-05 | DOWN |
| ANKRD10-IT1 | -4.701843506 | 1.50E-05 | DOWN |
| INHBA | -4.6816445 | 1.61E-05 | DOWN |
| COL8A1 | -4.674532511 | 1.66E-05 | DOWN |
| ITGA11 | -4.671356991 | 1.67E-05 | DOWN |
| SYTL2 | -4.660090121 | 1.74E-05 | DOWN |
| MFAP4 | -4.619815883 | 2.01E-05 | DOWN |
| PTN | -4.581243593 | 2.31E-05 | DOWN |
| COL6A3 | -4.561969617 | 2.48E-05 | DOWN |
| FPR3 | -4.555547211 | 2.53E-05 | DOWN |
| POSTN | -4.506632482 | 3.01E-05 | DOWN |
| TNC | -4.494515064 | 3.14E-05 | DOWN |
| CCL2 | -4.443817241 | 3.76E-05 | DOWN |
| FBLN5 | -4.431429181 | 3.93E-05 | DOWN |
| ABCC9 | -4.429576006 | 3.95E-05 | DOWN |
| CDH11 | -4.426828288 | 3.99E-05 | DOWN |
| ADH1B | -4.422261187 | 4.05E-05 | DOWN |
| OTTHUMG00000140109 | -4.419408376 | 4.10E-05 | DOWN |
| SAMHD1 | -4.364099394 | 4.97E-05 | DOWN |
| PRELP | -4.262758574 | 7.06E-05 | DOWN |
| THBS2 | -4.233764329 | 7.81E-05 | DOWN |
| SVEP1 | -4.176656595 | 9.49E-05 | DOWN |
| RNU7-75P | -4.175276329 | 9.54E-05 | DOWN |
| CD248 | -4.155242196 | 0.000102149 | DOWN |
| RGS4 | -4.143551531 | 0.000106301 | DOWN |
| PMP22 | -4.132858774 | 0.000110241 | DOWN |
| C7 | -4.114480679 | 0.000117345 | DOWN |
| CFH | -4.108046728 | 0.000119935 | DOWN |
| ABI3BP | -4.0991731 | 0.000123598 | DOWN |
| PROM1 | -4.097845941 | 0.000124155 | DOWN |
| MIR4275 | -4.089151691 | 0.000127864 | DOWN |
| IGLC7 | -4.079129406 | 0.000132273 | DOWN |
| CDH6 | -4.076217362 | 0.000133582 | DOWN |
| GPR34 | -4.019219468 | 0.000161851 | DOWN |
| OLFML2B | -4.013126936 | 0.000165193 | DOWN |
| COL14A1 | -3.999135317 | 0.000173123 | DOWN |
| VTRNA2-1 | -3.985536414 | 0.00018118 | DOWN |
| EDNRB | -3.963556218 | 0.000194973 | DOWN |
| COL3A1 | -3.956878886 | 0.000199359 | DOWN |
| SCARNA2 | -3.940222803 | 0.000210719 | DOWN |
| VCAN | -3.92459428 | 0.000221942 | DOWN |
| FMO3 | -3.917641364 | 0.000227118 | DOWN |
| VSIG4 | -3.886526724 | 0.000251735 | DOWN |
| SNORD99 | -3.870379673 | 0.000265501 | DOWN |
| RNA5SP217 | -3.855214434 | 0.000279086 | DOWN |
| PPP1R3C | -3.854334727 | 0.000279894 | DOWN |
| MS4A6A | -3.851669189 | 0.000282357 | DOWN |
| C1QC | -3.804851824 | 0.000329152 | DOWN |
| MMP7 | -3.803962583 | 0.000330109 | DOWN |
| SERPINF1 | -3.757586637 | 0.000383888 | DOWN |
| VCAN-AS1 | -3.742102858 | 0.000403642 | DOWN |
| C3 | -3.716675708 | 0.000438207 | DOWN |
| TNFRSF12A | -3.703023749 | 0.000457915 | DOWN |
| CCL11 | -3.675532759 | 0.000500203 | DOWN |
| CCL19 | -3.642197903 | 0.00055649 | DOWN |
| RNU6-79P | -3.636353469 | 0.000566962 | DOWN |
| LOC100131825 | -3.624290515 | 0.000589172 | DOWN |
| CCL18 | -3.551598863 | 0.000741611 | DOWN |
| CPA3 | -3.548992319 | 0.00074772 | DOWN |
| DAPL1 | -3.544978775 | 0.000757221 | DOWN |
| IGKV1D-16 | -3.535211483 | 0.000780823 | DOWN |
| LYVE1 | -3.524000097 | 0.000808778 | DOWN |
| CTSC | -3.502901486 | 0.000863984 | DOWN |
| PLVAP | -3.496493149 | 0.000881448 | DOWN |
| PLN | -3.4337961 | 0.001070938 | DOWN |
| IGKV1D-33 | -3.416767823 | 0.001128719 | DOWN |
| COLEC12 | -3.414193754 | 0.001137706 | DOWN |
| OTTHUMG00000035254 | -3.308258479 | 0.001572192 | DOWN |
| ITGB6 | -3.298165558 | 0.001620908 | DOWN |
| SNORD78 | -3.263786933 | 0.001797735 | DOWN |
| IGKC | -3.216785168 | 0.002069024 | DOWN |
| IGHV3-21 | -3.183516183 | 0.002283816 | DOWN |
| IGHA1 | -3.156924152 | 0.002470365 | DOWN |
| IGKV1-6 | -3.151387482 | 0.002510962 | DOWN |
| IGHJ5 | -3.13274545 | 0.002652293 | DOWN |
| IGKV1D-39 | -3.120769127 | 0.002746983 | DOWN |
| CLDN1 | -3.047011593 | 0.003403455 | DOWN |
| VCAM1 | -3.012484546 | 0.003758642 | DOWN |
| IGKV1D-27 | -2.99666651 | 0.003932636 | DOWN |
| IGKV1-9 | -2.979862135 | 0.00412567 | DOWN |
| NELL1 | -2.964255487 | 0.004312798 | DOWN |
| IGKV3D-15 | -2.92441683 | 0.004826806 | DOWN |
| KRT19 | -2.9036774 | 0.005116336 | DOWN |
| IGJ | -2.873391828 | 0.005568108 | DOWN |
| APOC1 | -2.866607249 | 0.005674245 | DOWN |
| IGKV1D-42 | -2.861694065 | 0.00575227 | DOWN |
| IGKV1-17 | -2.824846255 | 0.006369783 | DOWN |
| IGKV3-11 | -2.82214559 | 0.006417364 | DOWN |
| IGKV1-5 | -2.788900067 | 0.007030545 | DOWN |
| IGKV3D-7 | -2.788501179 | 0.007038219 | DOWN |
| IGHV3-30 | -2.708818938 | 0.008735131 | DOWN |
| IGLV6-57 | -2.688139807 | 0.009232946 | DOWN |
| IGKV2D-29 | -2.410649365 | 0.018928439 | DOWN |

**Table S6 74 common genes**

| **Name** | **change** |
| --- | --- |
| IGKV2D-29 | UP |
| PLCG2 | UP |
| IGF1 | UP |
| DEFA1B | UP |
| IGLV6-57 | UP |
| IGHV3-30 | UP |
| IGKV3D-7 | UP |
| IGKV1-5 | UP |
| IGKV3-11 | UP |
| IGKV1-17 | UP |
| IGKV1D-42 | UP |
| IGKV3D-15 | UP |
| IGKV1-9 | UP |
| VCAM1 | UP |
| IGKV1D-39 | UP |
| IGHJ5 | UP |
| IGKV1-6 | UP |
| IGHA1 | UP |
| IGHV3-21 | UP |
| SST | UP |
| IGKC | UP |
| COLEC12 | UP |
| IGKV1D-33 | UP |
| IGKV1D-16 | UP |
| CCL18 | UP |
| CCL19 | UP |
| CCL11 | UP |
| TNFRSF12A | UP |
| C3 | UP |
| KNG1 | UP |
| DEFB1 | UP |
| EDNRB | UP |
| APOM | UP |
| IGLC7 | UP |
| ANGPTL3 | DOWN |
| CCL2 | DOWN |
| RBP5 | DOWN |
| TNC | DOWN |
| PPBP | DOWN |
| PTN | DOWN |
| INHBA | DOWN |
| PTH1R | DOWN |
| IGHG4 | DOWN |
| FABP3 | DOWN |
| NR4A2 | DOWN |
| APLN | DOWN |
| CCL21 | DOWN |
| APOH | DOWN |
| TGFB2 | DOWN |
| CETP | DOWN |
| IGHJ1 | DOWN |
| EGF | DOWN |
| PI3 | DOWN |
| VEGFC | DOWN |
| ALB | DOWN |
| LTBP1 | DOWN |
| PTGDS | DOWN |
| ESM1 | DOWN |
| NR4A1 | DOWN |
| FPR2 | DOWN |
| AQP9 | DOWN |
| FPR1 | DOWN |
| GDF15 | DOWN |
| FCGR3B | DOWN |
| PTGS2 | DOWN |
| S100A9 | DOWN |
| S100A12 | DOWN |
| JUN | DOWN |
| S100A8 | DOWN |
| CXCR1 | DOWN |
| HSPA1B | DOWN |
| HSPA1A | DOWN |
| CXCR2 | DOWN |
| FOS | DOWN |

**Table S7 The results of functional enrichment analysis**

| **Category** | **ID** | **Term** | **P. val** |
| --- | --- | --- | --- |
| BP | GO:0006898 | receptor-mediated endocytosis | 2.63021E-19 |
| BP | GO:0006959 | humoral immune response | 4.06121E-18 |
| BP | GO:0030595 | leukocyte chemotaxis | 8.24186E-18 |
| BP | GO:0060326 | cell chemotaxis | 5.29028E-17 |
| BP | GO:0097529 | myeloid leukocyte migration | 2.90723E-15 |
| BP | GO:0030593 | neutrophil chemotaxis | 2.96822E-15 |
| BP | GO:1990266 | neutrophil migration | 2.38667E-14 |
| BP | GO:0071621 | granulocyte chemotaxis | 3.89749E-14 |
| BP | GO:0006958 | complement activation, classical pathway | 1.38738E-13 |
| BP | GO:0097530 | granulocyte migration | 2.92672E-13 |
| CC | GO:0019814 | immunoglobulin complex | 3.05743E-22 |
| CC | GO:0072562 | blood microparticle | 5.55034E-12 |
| CC | GO:0034774 | secretory granule lumen | 1.26416E-10 |
| CC | GO:0060205 | cytoplasmic vesicle lumen | 1.47227E-10 |
| CC | GO:0031983 | vesicle lumen | 1.58763E-10 |
| CC | GO:0031093 | platelet alpha granule lumen | 4.48633E-09 |
| CC | GO:0031091 | platelet alpha granule | 3.89777E-08 |
| CC | GO:0042571 | immunoglobulin complex, circulating | 3.30046E-07 |
| CC | GO:0062023 | collagen-containing extracellular matrix | 3.72587E-07 |
| CC | GO:0009897 | external side of plasma membrane | 1.93245E-05 |
| MF | GO:0048018 | receptor ligand activity | 7.16777E-12 |
| MF | GO:0030546 | signaling receptor activator activity | 8.35617E-12 |
| MF | GO:0003823 | antigen binding | 1.33042E-11 |
| MF | GO:0001664 | G protein-coupled receptor binding | 2.12998E-11 |
| MF | GO:0042379 | chemokine receptor binding | 4.51467E-09 |
| MF | GO:0008083 | growth factor activity | 4.73388E-09 |
| MF | GO:0048020 | CCR chemokine receptor binding | 1.35862E-08 |
| MF | GO:0008009 | chemokine activity | 1.7598E-08 |
| MF | GO:0050786 | RAGE receptor binding | 4.35715E-08 |
| MF | GO:0005125 | cytokine activity | 1.18599E-07 |
